# Impaired anaplerosis is a major contributor to glycolysis inhibitor toxicity in glioma

**DOI:** 10.1186/s40170-021-00259-4

**Published:** 2021-06-25

**Authors:** Sunada Khadka, Kenisha Arthur, Yasaman Barekatain, Eliot Behr, Mykia Washington, Jeffrey Ackroyd, Kaitlyn Crowley, Pornpa Suriyamongkol, Yu-Hsi Lin, Cong-Dat Pham, Rafal Zielinski, Marissa Trujillo, James Galligan, Dimitra K. Georgiou, John Asara, Florian Muller

**Affiliations:** 1 Department of Cancer Systems Imaging, The University of Texas MD Anderson Cancer Center, Houston, TX USA; 2 Department of Cancer Biology, The University of Texas MD Anderson Cancer Center, Houston, TX USA; 3 MD Anderson UT Health Graduate School of Biomedical Sciences, Houston, TX USA; 4 Department of Experimental Therapeutics, The University of Texas MD Anderson Cancer Center, Houston, TX USA; 5 Department of Pharmacology and Toxicology, College of Pharmacy, University of Arizona, Tucson, AZ USA; 6 Department of Medicine, Beth Israel Deaconess Medical Center and Harvard Medical School, Boston, MA USA; 7 SPOROS Bioventures, Houston, TX USA

**Keywords:** Cancer metabolism, Anaplerosis, Collateral lethality, Glycolysis, Glutaminolysis, Enolase inhibitor, POMHEX, CB-839

## Abstract

**Background:**

Reprogramming of metabolic pathways is crucial to satisfy the bioenergetic and biosynthetic demands and maintain the redox status of rapidly proliferating cancer cells. In tumors, the tricarboxylic acid (TCA) cycle generates biosynthetic intermediates and must be replenished (anaplerosis), mainly from pyruvate and glutamine. We recently described a novel enolase inhibitor, HEX, and its pro-drug POMHEX. Since glycolysis inhibition would deprive the cell of a key source of pyruvate, we hypothesized that enolase inhibitors might inhibit anaplerosis and synergize with other inhibitors of anaplerosis, such as the glutaminase inhibitor, CB-839.

**Methods:**

We analyzed polar metabolites in sensitive (*ENO1*-deleted) and resistant (*ENO1*-WT) glioma cells treated with enolase and glutaminase inhibitors. We investigated whether sensitivity to enolase inhibitors could be attenuated by exogenous anaplerotic metabolites. We also determined the synergy between enolase inhibitors and the glutaminase inhibitor CB-839 in glioma cells in vitro and in vivo in both intracranial and subcutaneous tumor models.

**Results:**

Metabolomic profiling of *ENO1*-deleted glioma cells treated with the enolase inhibitor revealed a profound decrease in the TCA cycle metabolites with the toxicity reversible upon exogenous supplementation of supraphysiological levels of anaplerotic substrates, including pyruvate. *ENO1-*deleted cells also exhibited selective sensitivity to the glutaminase inhibitor CB-839, in a manner rescuable by supplementation of anaplerotic substrates or plasma-like media Plasmax^TM^. In vitro, the interaction of these two drugs yielded a strong synergistic interaction but the antineoplastic effects of CB-839 as a single agent in *ENO1*-deleted xenograft tumors in vivo were modest in both intracranial orthotopic tumors, where the limited efficacy could be attributed to the blood-brain barrier (BBB), and subcutaneous xenografts, where BBB penetration is not an issue. This contrasts with the enolase inhibitor HEX, which, despite its negative charge, achieved antineoplastic effects in both intracranial and subcutaneous tumors.

**Conclusion:**

Together, these data suggest that at least for *ENO1*-deleted gliomas, tumors in vivo—unlike cells in culture—show limited dependence on glutaminolysis and instead primarily depend on glycolysis for anaplerosis. Our findings reinforce the previously reported metabolic idiosyncrasies of in vitro culture and suggest that cell culture media nutrient composition more faithful to the in vivo environment will more accurately predict in vivo efficacy of metabolism targeting drugs.

**Supplementary Information:**

The online version contains supplementary material available at 10.1186/s40170-021-00259-4.

## Background

Tumor cells must undergo significant alterations in their metabolic pathways to support their bioenergetic and biosynthetic demands in their mostly nutrient- and oxygen-deficient microenvironment [1–3]. One such metabolic adaptation is the Warburg phenomenon, a bioenergetically inefficient process characterized by an increased reliance of cancer cells on glycolysis for ATP production, making glycolysis a targetable metabolic vulnerability in diverse cancers [4, 5]. However, therapeutically targeting glycolysis specifically in cancer cells remains aspirational because glycolysis is an indispensable metabolic pathway for some nonmalignant cells [6]; thus, blockade of glycolysis is incompatible with life. However, synthetic lethal interactions supported by glycolysis can be exploited to specifically target cancer cells. Building on this idea, we conceived the framework of *collateral lethality*, whereby incidental loss of passenger metabolic genes—genes that are lost along with driver tumor suppressor genes—can be exploited therapeutically by targeting the redundant isoforms of the passenger genes [7, 8]. Using this concept of collateral lethality, we validated that in a subset of gliomas with 1p36 deletions, passenger deletion of the glycolytic gene *ENO1* selectively renders cancer cells sensitive to inhibition of the redundant isoform *ENO2* [8]. Thus, to exploit this therapeutic opportunity in this subset of tumors, we developed a specific inhibitor of enolase, HEX and a pro-drug thereof, POMHEX. Both POMHEX and HEX exerted dramatic antineoplastic effects on *ENO1*-deleted tumor cells in culture and in *ENO1*-deleted xenograft tumors. Metabolomic and biochemical data strongly indicated both specific and dose-dependent inhibition of glycolysis by these enolase inhibitors [9].

Besides glucose, cancer cells also exhibit a predilection for glutamine, a nonessential amino acid that cancer cells consume disproportionately to support their biosynthetic requirements [10, 11]. Multiple studies have shown that cancer cells are “addicted” to glutamine, at least in vitro [10, 11]. Therefore, glutamine metabolism has emerged as another promising therapeutic target, and telaglenastat (CB-839), a glutaminase inhibitor targeting glutamine metabolism is currently being investigated in randomized clinical trials against a range of malignancies (https://www.clinicaltrials.gov/, NCT03428217, NCT04265534) [12].

Pyruvate derived from glycolysis and glutamate derived from glutaminolysis converge to replenish tricarboxylic acid (TCA) cycle intermediates at different steps. The TCA cycle is strategically situated at the center of cellular metabolism, where it serves as an anabolic hub for the synthesis of macromolecules such as fatty acids, cholesterol, and amino acids that are crucial to support rapidly growing tumors. Carbon atoms are constantly drained from the TCA cycle for biosynthetic reactions and for CO2 release by the NAD-dependent dehydrogenases to supply reducing equivalents to oxidative phosphorylation. The TCA cycle must thus be constantly replenished by carbon atoms, a process termed *anaplerosis* [13]. The coupling of anaplerosis and cataplerosis—the removal of TCA cycle intermediates—is critical to maintaining the balance of the TCA cycle metabolites that are essential for supporting crucial biosynthetic reactions. Additionally, this coupling also ensures that redox equilibrium and ATP production via oxidative phosphorylation are maintained. The current dogma in biochemistry is that the main anaplerotic substrates are pyruvate (from glucose) and glutamate (from glutamine), with lesser contributions from precursors of propionyl-CoA, such as odd-chain fatty acids, amino acids, and C5 ketone bodies that feed into succinate [14]. With a view to exploiting both the increased dependence of cancer cells on glucose and glutamine for their growth and the roles of these molecules in TCA cycle anaplerosis, we surmised that impairing the use of these two key nutrients can be exploited as a therapeutic strategy to induce bioenergetic and anaplerotic nutrient stress and suppress tumor growth.

Using the collateral lethality paradigm, we previously reported that a subset of glioblastoma tumors with collateral homozygous deletion of *ENO1* along with the 1p36 tumor suppressor locus is extraordinarily sensitive to the inhibition of *ENO1*’s redundant paralog *ENO2* [7, 8]. Here, we show that a major mechanism of toxicity of glycolysis inhibition by the enolase inhibitor POMHEX is depletion of TCA cycle intermediates via inhibition of the formation of anaplerotic pyruvate from glucose. In support of this model, metabolomic data indicate significant decreases in the TCA cycle intermediates malate and fumarate in response to enolase inhibition. We show that enolase inhibitor toxicity can be partially rescued by high-dose exogenous pyruvate supplementation in the media. Furthermore, inhibition of anaplerotic reactions through glutaminase by the clinical-grade glutaminase inhibitor CB-839 is synergistically toxic with the enolase inhibitor POMHEX, in a manner also modulated by the availability of exogenous pyruvate. Furthermore, CB-839 displays selective toxicity toward cells with homozygous deletions of *ENO1* but not isogenic rescued cells even in the absence of the enolase inhibitor, and this toxicity can be completely alleviated by pyruvate supplementation in the media. The in vitro data strongly indicated that anaplerosis could be targeted for cancer therapy, provided that one arm (glycolysis or glutaminolysis) is inactivated or deficient in a specific cancer cell. However, despite mechanistically promising in vitro data, the synergistic effect endowed by the enolase and glutaminase inhibitors could not be entirely recapitulated in vivo in both orthotopic (intracranial) and subcutaneous tumor models. Our observation that cancer cells in culture may depend on both glycolysis and glutaminolysis, with glutamine oxidation being essentially dispensable for tumor sustenance in vivo, echoes previously reported discrepancies in tumor metabolism in in vitro vs. in vivo system [15, 16].

## Methods

### Cell lines and enolase and glutaminase inhibitor toxicity testing in vitro

The cell lines used in the experiments were LN319 (CVCL_3958, glioblastoma), H423/D423-MG (CVCL_1160, glioblastoma), H502/D502-MG (CVCL_1162, glioblastoma), U-343MG (CVCL_S471, glioblastoma), and ENO1 isogenic rescue, D423 ENO1. LN319 has been previously described and is *ENO1* wild-type; it has been identified as a subclone of LN992 [17], but for our experiment—as an *ENO1* wild-type control—this was considered acceptable. The H423/D423-MG cell line, referred to as D423 here, features a 1p36 homozygous deletion encompassing *ENO1* [8]. The H502/D502-MG cell line, referred to as D502 throughout the paper, has a 1p36 homozygous deletion but with ENO1 excluded and not deleted. Both D423 and D502 were generously provided by D. Bigner. U343-MG is heterozygous for *ENO1* and has been described previously [8]. D423 ENO1 is an isogenic cell line generated by our lab for previous experiments and engineered for constitutive and ectopic expression of ENO1 [18]. All cell lines were cultured at 37 °C in a 5% CO_2_ atmosphere in Dulbecco’s modified Eagle medium (DMEM). The DMEM used contained 4.5 g/L glucose, 110 mg/L pyruvate, and 584 mg/L glutamine (Cellgro/Corning #10-013-CV) and contained 10% fetal bovine serum, 1% penicillin/streptomycin, and 0.1% Amphotericin B. Cell lines were validated by the Characterized Cell Line Core (CCLC) at The University of Texas MD Anderson Cancer Center using the Promega 16 High Sensitivity STR KIT. Short tandem repeat profiles were compared to the CCLC database and external cell databases (ATCC/DSMZ/RIKEN/JCRB). Cell lines were tested on a regular basis for mycoplasma contamination using the MycoAlert PLUS detection kit (Lonza #LT07-118).

To test the sensitivity of each cell line to enolase and glutaminase inhibitors, we seeded 2000 cells per well in 96-well plates in appropriate media. After incubating for 24 h, cells in columns 3-10 of the 96-well plate were treated with fresh media containing an enolase inhibitor in a twofold dilution series. Columns 1, 2, 11, and 12 were given fresh media and left as controls. For tests of combination treatments with POMHEX and CB-839, a uniform dose of 500 nM CB839 was administered in rows 3-12, while POMHEX dilution treatments were administered in rows 3-10. After 7 days, cells were washed with phosphate-buffered saline (PBS) and fixed in 10% formalin. Fixed cells were stained with 0.05% crystal violet and extracted with 10% acetic acid. Cell viability was quantified by spectrophotometric absorption at 595 nm using a microplate reader.

### CellTitre-Glo® for ATP measurement

Cells were seeded in 96-well plates as described previously, and treated in serial dilution of POMHEX from 10 μM to 78 nM in 100 μl of normal Plasmax^TM^ medium or Plasmax^TM^ medium supplemented with 5 mM pyruvate for 24 h. Row A and H were left empty to measure background luminescence. After 24 h of drug exposure, cells were incubated at the room temperature before adding cell titer glo reagent. One volume of CellTitreGlo® reagent (Promega, #G9242) was added to each well, and the contents were mixed on an orbital shaker for 2 min to allow cell lysis to occur. After 10 min of incubation at room temperature, the contents were transferred to opaque walled (Falcon #353296) 96-well plates and luminescence was recorded in a microplate reader.

### Seahorse analysis

The mitochondrial respiratory capacity of *ENO1* deleted D423 and *ENO1* reconstituted D423 *ENO1* cells was measured using the Seahorse XFp Mito Stress Test Kit (Agilent, #103707-100) according to the manufacturer’s protocol. A total of 40,000 cells per well were plated in regular DMEM supplemented with 10% FBS and 1% penicillin–streptomycin on a 96-well Seahorse microplate 24 h before the experiment. The following day, an hour before the experiment, DMEM medium was discarded and the plate was once washed with Seahorse medium (Agilent, #103680-100), while ensuring that the cells were still adherent on the plate. One hundred eighty microliters of Seahorse medium supplemented with 2 mM l-glutamine, 10 mM glucose with or without 1 mM pyruvate was added to the cells (wells 1-6 were treated with Seahorse medium without pyruvate and wells 7-12 were treated with Seahorse medium with 1 mM pyruvate). Cells were then incubated at 37 °C (low CO_2_) for 1 h. The Seahorse cartridge was hydrated overnight in the Seahorse calibrating solution (Agilent, #100840-000). On the day of the experiment, the cartridge was loaded with 20 μl of the drugs as follows: POMHEX—PORT A (POMHEX dilution from 1 μM to 0.062 μM), oligomycin (1 μM)—PORT B and FCCP (1 μM)—PORT C and rotenone/antimycin (0.5 μM)—PORT D. The concentrations of the drugs in the ports were adjusted to achieve the listed final concentrations. The OCR and ECAR were measured using the Seahorse XF Analyzer. POMHEX was added 60 min before mitochondrial stressors were added and 3 sets of ECAR and OCR measurements were taken for each treatment. OCR and ECAR values were normalized to the baseline values.

### Experimental animals

All in vivo experiments with mice were approved by MD Anderson’s Institutional Animal Care and Use Committee (IACUC) and performed in an AAALAC-accredited facility at MD Anderson. Mice used in this experiment were immunocompromised female nude *Foxn1*^nu/nu^ mice bred at the Experimental Radiation Oncology Breeding Core at MD Anderson.

### Establishment of orthotopic intracranial tumors

Intracranial glioma tumors were established in mice aged 4 to 6 months. First, bolts were inserted into the skulls of the mice by drilling a hollow plastic screw into the skull [19]. Mice were given 2 weeks to recover and monitored for signs of morbidity. Next, using a Hamilton syringe, 200,000 glioma cells were injected into the brain of the mice through the bolt. Both intracranial bolt implantation and tumor injection were performed by the MD Anderson Intracranial Injection Fee-for-Service Core (Dr. Fred Lang, Director). Throughout the experiment, any animals exhibiting severe neurological morbidities were euthanized according to IACUC regulations.

### In vivo tumor volume measurement

Prior to undergoing magnetic resonance imaging (MRI), the mice were briefly anesthetized with isoflurane. Throughout the imaging protocol, the animal’s body temperature was maintained with a heating blanket, and a stereotactic holder was used to restrain the mouse and hold its head in place. The animals’ heart and breathing rates were monitored throughout the imaging procedure. Weekly T2-weighted MRI scans of intracranial tumors were performed with a 7T Biospec USR70/30 MRI system (Bruker Biospin MRI, Billerica, MA) in MD Anderson’s Small Animal Imaging Facility (SAIF).

To center the image, a low-resolution axial scan was first taken. After calibration, one high-resolution coronal scan was taken with a slice thickness of 0.750 mm and a slice spacing of 1.000 mm. Next, two high-resolution axial scans, each offset from the other by 0.500 mm, were taken, with a slice thickness of 0.500 mm and slice spacing of 1.000 mm. The offset of 0.500 mm was chosen to allow better coverage of the tumor, as the scans individually had noncontiguous slices. For each scan, the number of slices varied based on tumor size.

MRI scans were analyzed with the open-source software 3D slicer (v4.10, https://www.slicer.org) by 4 independent members of our labs [20]. For each slice, the Draw tool in the Editor module was used to manually select tumor tissue. Areas of edema and concave regions of the tumor where the bolt was implanted were excluded from the selections. Using the Label Statistics module, tumor volumes from each scan were calculated automatically by summing the selected pixel area from each slice, converting it to cm^2^ in accordance with the DICOM metadata, and multiplying it by the slice spacing of 1.000 mm. Each lab member then calculated a final volume for the entire MRI series as the mean of the 3 volumes (1 coronal, 2 axial) they determined. For final data analysis purposes, the volume for each MRI series was the average of the volumes calculated by the 4 lab members.

### Establishment of subcutaneous tumors

Subcutaneous tumors were established in mice aged 2-4 months. *ENO1* deleted cells were first trypsinized and washed with PBS twice. The cells were then resuspended in PBS and mixed with MatrigelBD in a 1 to 1 ratio and left on ice to prevent Matrigel solidification. Two hundred microliters (5 million cells total) of Matrigel-cell suspension was then administered to each anesthetized mouse on its right flank. The mice were monitored for a week for any sign of inflammation.

Tumors were measured three times a week using Vernier’s calipers. Two dimensions were measured, and the volume was determined as the product of two dimensions and the average of two dimensions. When the tumors reached approximately 150 mm^3^, the mice were grouped into different treatment groups for drug administration.

### Xenografted mouse drug treatment with HEX and CB-839

A solution of HEX in ddH_2_O (250 mg/ml) was made and adjusted to 7.2-7.4 pH, then sterile-filtered and stored at −20 °C. HEX (300 mg/kg) was injected subcutaneously into mice in drug volumes adjusted according to their weight.

CB-839 (20 mg/ml) formulation was provided by Calithera Biosciences along with the vehicle control. Both CB-839 and vehicle controls were aliquoted and frozen in −20 °C and administered 200 mg/kg BID by oral gavage. Per Calithera’s instructions, the thawed drug was used one-time only and the left-over drug was discarded.

### Polar metabolite profiling of cells and extracted tumors

To profile polar metabolites, we used the Johan Asara Metabolomics Platform at the Beth Israel Deaconess Medical Center (BIDMC) [21]. Polar metabolite samples were analyzed by liquid chromatography-tandem mass spectrometry using a 5500 QTRAP hybrid triple quadrupole mass spectrometer (SCIEX) coupled to a high-performance liquid chromatography column (Shimadzu) with an amide hydrophilic interaction chromatography column (waters; pH = 9.0 at 400 mL/min). Selection reaction monitoring with polarity switching tracked a total of 300 polar metabolites of interest from both cells and tumor tissue. Q3 peaks were integrated using the MultiQuant 2.1 software.

Glioma cells grown in 10 cm dishes were harvested at roughly 90% confluency. First, the medium was removed, and the cells were washed once with cold PBS. Then, 4 mL of 80% methanol precooled to −80 °C was added, and the cells were incubated for 20 min on −80 °C dry ice. Cells were harvested using a scraper, and lysates were collected into precooled tubes. Cell lysates were spun down for 5 min at 18,000×*g* at 4 °C to precipitate cell debris and nonpolar metabolites. The polar metabolites collected into the supernatant, which was aliquoted into 1.5-mL Eppendorf tubes and dried with a Thermo Fisher SpeedVac. The concentrated and dried polar metabolites were then sent for profiling to the BIDMC Metabolomics Platform.

Tumors were harvested when they grew to approximately 1000 mm^3^. First, animals were euthanized under a standard IACUC protocol, usually after 4-6 h of final drug dose administration. Subcutaneous tumors were then extracted, weighed, and cut in half. One half was promptly snap-frozen in liquid N_2_ for further metabolomic analysis, while the other half was fixed in 10% formalin for immunohistochemical analyses. Snap-frozen tumor chunks were cut into pieces weighing roughly 50 mg and transferred to chilled microcentrifuge tubes (Fisher Scientific, Cat. 02-681-291) containing Qiagen steel beads. After 1 mL of −80 °C, 80% methanol was added to tubes; tumor tissue was bead-mill homogenized at 28 Hz for multiple durations of 45 s using a Qiagen TissueLyser. Eighty percent methanol was then added to the homogeneous tumor lysate to reach a final 50 mg/2 mL composition. Tumor lysates were then incubated for 15 min on dry ice and homogenized one more for 1 min with a vortex mixer. Samples were then promptly centrifuged for 5 min at 14,000×*g* at 4 °C. The supernatants containing polar metabolites were submitted to BIDMC for analysis.

### Isotope labeling and mass-spec analysis

Cells were treated with DMSO or 75 nM POMHEX in pyruvate free DMEM or DMEM supplemented exogenously with U-13C pyruvate (Cambridge Isotope Laboratories #CLM-2440-0.5). After 12 h, metabolites were extracted in 80% cold methanol and dried in speed vac. Dried metabolites were resuspended in 150 μL of ice-cold 80:20 MeOH:H_2_O containing 5 nmol of CEL-d_4_. Samples were briefly sonicated into solution and spun down for 10 min (4 °C, 14,000×*g*). Metabolites were chromatographed using a Shimadzu LC system equipped with a 2.1 mm × 100 mm, 3.5 μm particle diameter XBridge Amide column (Waters, Milford, MA) at a flow rate of 0.40 mL/min. Buffer A (95% (v/v) ddH_2_O, 5% (v/v) ACN, 20 mM NH_4_OH, 20 mM NH_4_OAc) and Buffer B (ACN) were chromatographed as follows: 0.5 min, 95% B; 15.0 min, 50% B; 17.0 min, 2% B; 19.0 min, 2% B; 19.5 min, 95% B. The column was equilibrated at 95% B for 5 min between runs. Multiple reaction monitoring was performed in negative ion mode using an AB SCIEX 4500 QTRAP with the parameters defined in the table below. All compounds were quantified against the IS (CEL-d_4_) and normalized to total protein

### Immunohistochemistry

Mice were euthanized either when their subcutaneous tumors reached 1000 mm^3^ or earlier if they exhibited any morbidities. The tumors were harvested and promptly fixed in 10% paraformaldehyde. The fixed tumors were then submitted to the Veterinary Pathology Core at MD Anderson, where tissues were dehydrated, embedded in paraffin, and sectioned. We dried the sectioned tissue slides overnight at 60 °C before deparaffinization with xylene. Deparaffinized slides were rehydrated via a dilution series of aqueous ethanol. For antigen retrieval, slides were boiled in citrate buffer for 10 min and cooled for 30 min. Slides were then incubated for 1 h in 2% goat serum for blocking. Blocked slides were incubated evenly with primary antibodies diluted to 1:1000 in PBS containing 2% goat serum for 24 h at 4 °C. The primary antibodies used were anti-phospho S10 histone H3 (rabbit anti-phospho histone H3 (S10) IHC antibody, affinity purified; Bethyl Laboratories, IHC-00061) and monoclonal anti-cleaved caspase 3 rabbit (cleaved caspase-3 (Asp175) (5A1E) rabbit mAb; CST# 9664T, Cell Signaling Technology). Following 3-5-min washes in PBS with shaking, slides were incubated with 1× goat anti-rabbit Poly HRP secondary antibody (Thermo Fisher, #B40962) for 30 min and then washed in PBS with Tween (3 × 5 min) with shaking before developing. Sections were developed using EnzMet (Nanoprobes # 6001-30 ML, yields a black stain) that were counterstained with hematoxylin and eosin. Stained slides were mounted using Thermo Scientific Cytoseal 60 and Ultra Microscope Cover Glass and dried for 24 h at room temperature.

### Quantification of PH3 and CC3 signals

Aperio Images scope (Leica Biosystem) was used to snap pictures of the slides. Pictures of 10× (×100 with objective) sections of 1.6×10^6^ μm^2^ surface area were taken and the black stains for PH3 and CC3 were counted. Results are expressed as positive nuclei per 10× section.

### Mass-spec detection of CB-839 in mouse and human plasma

A stock solution of CB-839 in DMSO was made and aliquots were administered in mice. The human plasma control was prepared from a commercially available powder (Sigma Aldrich, LOT# SLBX8880). The powder was dissolved in ddH_2_O to which an aliquot of CB-839 was added to make a final concentration of 50 μM. All samples except the DMSO control were extracted with ethyl acetate from their respective sources and diluted in MeOH (final volume 1 mL). Mass spectra were obtained on a Waters Zspray^TM^ using an electrospray ion source (ESI+/−) via direct infusion and processed with MassLynx (V4.2 SCN 985).

### Statistical analysis

Statistical analyses reported in this study were performed using either Microsoft Excel or Graph Pad Prism 8. Unpaired Student’s test and 1- or 2-way ANOVA were used where appropriate. Tukey’s post hoc analysis was used to determine statistical significance following ANOVA. *P*<0.05 was used as a threshold to determine statistical significance.

## Results

### Enolase inhibition represses oxidative phosphorylation, depletes TCA cycle metabolites, and induces bioenergetic collapse

**Fig. 1:**
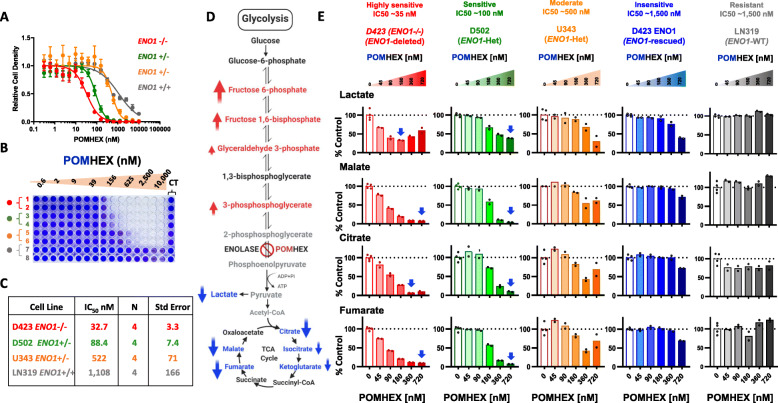
Metabolomic profiling of enolase-inhibitor treated glioma cell lines indicates a profound disruption in anaplerosis, which correlates with the sensitivity. **a–c** Sensitivity of glioma cells to the enolase inhibitor is reflected by their *ENO1* status*.* **a** Dose-response curves of *ENO1* homozygously deleted (D423; red, *N*=4), *ENO1*-heterozygously deleted (D502; green, *N*=4; U343; orange, *N*=4), and *ENO1* wild type (LN319; grey *N*=4) cells treated with the enolase inhibitor POMHEX at the indicated doses. Error bars represent the standard error of mean. **b** After 5 days of treatment, the cells were fixed in 10% formalin and stained with crystal violet dye to measure the terminal cell density. The terminal cell density is expressed relative to the untreated controls. **c** A representative table with the IC50 values of POMHEX across different cell lines strongly indicates that *ENO1* homozygously deleted cells are selectively sensitive, while *ENO1* heterozygotes display intermediate sensitivity to POMHEX. **d-e** Enolase inhibitor causes a profound disruption in the TCA cycle. Cells were treated with varying concentrations of POMHEX in DMEM media and the metabolites were extracted in 80% cold methanol after 72 h of drug treatment. The extracted metabolites were subjected to metabolomic analysis by mass-spectroscopy. **d** Schematic showing the glycolytic and TCA cycle metabolites that are altered by POMHEX treatment. Red arrows indicate metabolites that are elevated, while blue arrows indicate metabolites that are decreased in response to POMHEX. **e** Lactate levels are shown as an indicator of glycolysis inhibition in response to the enolase inhibitor POMHEX. Two TCA cycle intermediates citrate and malate are shown as representative metabolites in the TCA cycle that are altered in a dose dependent manner as a result of enolase inhibition. The effects of enolase inhibition on TCA cycle metabolites correlate with the levels of ENO1 in different cell lines, with the *ENO1*-homozygously deleted cells exhibiting the most profound change, followed by *ENO1* heterozygous cells showing intermediate effect while *ENO1* intact wild type cells sustaining no significant effect (see Supplemental figure S1 and S2 for a full panel of glycolytic and TCA cycle metabolites)

We previously demonstrated that glioma cells with *ENO1* passenger deletions are selectively susceptible to inhibition of *ENO1*’s redundant paralog *ENO2* through the collateral lethality paradigm [8]. To test the therapeutic utility of this concept in tumors with *ENO1* deletions, we developed small-molecule inhibitors of the glycolytic enzyme enolase. HEX, a phosphono-hydroxamate, as well as its prodrug, pivaloyloxymethyl (POM)-adduct POMHEX, both potently inhibit glycolysis and display fourfold greater specificity against ENO2 than ENO1 [9]. In the current study, we found that glioma cell lines that have either homozygous (D423) or heterozygous (D502, U343) deletions of *ENO1* are selectively more sensitive to the enolase inhibitor POMHEX than are *ENO1* rescued (D423 *ENO1*) and wild-type cells (LN319) (Fig. 1 a-c). The dose-response curves and the half maximal inhibitory concentration (IC50) data showed that the enolase inhibitor sensitivity of the cancer cell lines correlated with their *ENO1* status. Cells with *ENO1* homozygous deletions incurred the most significant toxicity from POMHEX, followed by cells with *ENO1* heterozygous deletions and *ENO1* wild type cells (Fig. 1 a-c). Notably, D502 and U343 cells, despite both having *ENO1* heterozygous deletions, displayed differential sensitivities to inhibition of enolase. We surmised that differences in the basal levels of carboxylesterase and phosphodiesterase, the enzymes that cleave the POM adduct from POMHEX, may explain the differences in enolase inhibitor sensitivity between D502 and U343.

Next, we sought to elucidate the mechanistic basis of the toxicity induced by enolase inhibition by performing comprehensive metabolomic profiling. Cells treated with POMHEX showed elevation in the levels of metabolites upstream of enolase and a concurrent decrease in lactate, indicating effective target engagement by the inhibitor (Fig. 1 d-e, and Supplemental Figure S1 for other glycolytic intermediates). Additionally, across glioma cell lines treated with POMHEX in vitro, we consistently observed dose-dependent depletion of major TCA cycle metabolites such as citrate, fumarate, and malate (Fig. 1 d-e, and Supplemental Figure S2 for other TCA cycle metabolites). Interestingly, the extent of the disruption in the TCA cycle metabolites corresponded with the sensitivity of the cell line to the inhibitor. This was particularly evident in D502 and U343 cells; D502 cells were more sensitive than U343 cells to POMHEX (Fig. 1 a and b) and displayed more substantial depletion of TCA cycle intermediates in response to POMHEX than did U343 cells (Fig. 1 e, and Supplemental Figure S2). Abrogation of pyruvate formation by enolase inhibition depletes a prominent source of carbon atoms that fuels the TCA cycle, effectively diminishing the levels of TCA cycle intermediates. Thus, these findings suggest that a proximal cause of toxicity by POMHEX is the depletion of the anaplerotic substrate pyruvate.

**Fig. 2:**
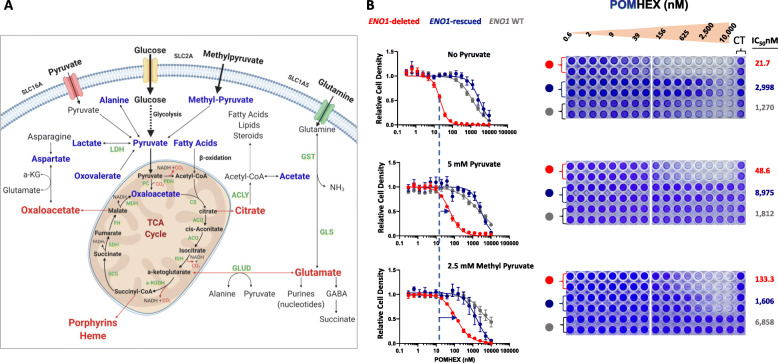
Exogenous supplementation of selected anaplerotic substrates mitigates the toxicity of enolase inhibition. **a** Schematic representing different cellular metabolites that converge to replenish the TCA cycle carbon atoms. Exogenously supplemented anaplerotic substrates (alanine, acetate, aspartate, fatty acids, lactate, methylpyruvate, pyruvate, oxaloacetate, and oxovalerate) are indicated in blue, while cataplerotic substrates are indicated in red. **b** Dose response curves of *ENO1-*deleted, *ENO1-*rescued and *ENO1* wild type cells to POMHEX in pyruvate free DMEM (*N*=12) and DMEM exogenously supplemented with 5 mM pyruvate (*N*=6) and 2.5 mM methyl pyruvate (*N*=4). Cells were seeded in 96-well plates in pyruvate free DMEM or DMEM supplemented with the anaplerotic substrates and treated with serial dilutions of POMHEX. Crystal violet staining was performed to measure the terminal cell density and assess the effect of POMHEX and the degree of rescue of POMHEX toxicity by exogenous supplementation of anaplerotic substrates. Cell density is expressed relative to the untreated controls. A shift in IC50 (blue horizontal arrows) indicates alleviation of POMHEX toxicity by addition of exogenous anaplerotic substrates. IC50 of POMHEX for each cell line in different medium condition is indicated. (see Supplemental Figure S4 and S5 for a panel of anaplerotic substrates)

Because the depletion of TCA cycle intermediates was found to be the most prominent metabolic effect of enolase inhibition with POMHEX and a strong correlate of cells’ sensitivity to enolase inhibition, we sought to determine whether exogenous supplementation with physiological, supraphysiological, or artificial exogenous anaplerotic substrates could modulate enolase inhibitor toxicity in vitro. Different cellular metabolites funnel into the TCA cycle to replenish the carbon atoms at different steps (Fig. 2 a); thus, we performed in vitro rescue experiments in pyruvate-free DMEM with a panel of anaplerotic substrates (Fig. 2, Table 1 and Supplemental Figure S3, 4). We found that the medium supplemented with physiological levels of pyruvate (100 μM) only minimally attenuated POMHEX toxicity (data not shown), but medium with supraphysiological levels of pyruvate (5 mM) resulted in a 2.5-fold increase in IC50 of POMHEX in cells with *ENO1* homozygous or heterozygous deletions (Fig. 2 b, and Supplemental Figure S3). Consistently, methyl-pyruvate, a synthetic cell-permeable pro-metabolite of pyruvate, and oxaloacetate, a TCA cycle metabolite, dramatically rescued POMHEX toxicity, as evidenced by a sixfold and a fourfold increase, respectively, in the IC50 of POMHEX in *ENO1*-deleted cells (Fig. 2 b, and Supplemental Figure S4C). Notably, dimethyl 2-oxoglutarate, a synthetic cell permeable analog of α-ketoglutarate and a direct anaplerotic substrate (Supplemental Figure S4B), provided maximal rescue to POMHEX toxicity. We also repeated the anaplerotic substrate rescue experiments in physiological Plasmax^TM^ medium (Supplemental Figure S5) [22] and found that supraphysiological levels of pyruvate (5 mM) and dimethyl 2-oxoglutarate (5 mM) provided comparable and maximal rescue in POMHEX-treated cells in Plasmax^TM^ medium as well (Supplemental Figure S5 A-H).

**Table 1:** Representing the IC50 values of *ENO1-*deleted cells treated with POMHEX in pyruvate free DMEM or DMEM exogenously supplemented with different anaplerotic substrates at the indicated concentrations. IC50 values in italic indicate metabolites that confer partial or complete rescue to POMHEX toxicity. Human plasma metabolite concentrations are indicated where relevant, based on human metabolome database (HMD). Pyruvic acid (HMDB0000243), lactic acid (HMDB0000190), acetate, (HMDB0000042) alanine, (HMDB0000161), aspartate (HMDB0000191), and oxovalerate (HMDB0001865)

Anaplerotic substrates	D423	N	Concentrations
	***ENO1*** deleted		in Human Blood (μM)
Pyruvate Free	22	12	
100 μM Pyruvate	28	6	35
1 mM Pyruvate	*51*	6	35
5 mM Pyruvate	*49*	6	35
2.5 mM Methyl Pyruvate	*133*	4	N/A
5 mM dimethyl 2-oxoglutarate	*189*	2	N/A
5 mM Lactate	19	4	1,118-1,860
5 mM Acetate	19	2	27-64
5 mM Alanine	11	2	427
2.5 mM Aspartate	29	2	8-20
1X B27	36	2	N/A
1X Fatty Acid	39	2	N/A
2.5 mM Oxaloacetate	*87*	4	N/A
2.5 mM Oxovalerate	*60*	4	13

Since pyruvate is in equilibrium with NAD+ through the lactate dehydrogenase reaction (LDH), exogenous pyruvate is expected to alter the NAD+/NADH redox balance. We interrogated whether NAD+ regeneration by exogenously supplemented pyruvate to lactate conversion has a stimulatory effect on glycolysis. To distinguish which of the two mechanisms, NAD+ regeneration or anaplerosis, takes precedence in providing pyruvate mediated POMHEX toxicity relief, we tested the rescue potential of α-ketobutyrate (AKB), a pyruvate mimetic that can undergo LDH reaction to regenerate NAD+, but cannot serve as a mitochondrial substrate for the TCA cycle and ATP production [23]. We found that AKB provides minimal rescue to enolase inhibition in *ENO1* deleted cells and is nowhere as effective as pyruvate at the same concentration (Supplemental Figure S5A-E). Next, we also performed Seahorse Mito Stress Test on POMHEX-treated cells with and without pyruvate supplementation. We found that POMHEX treatment causes a decrease in both extracellular acidification rate (ECAR) and oxygen consumption rate (OCR) as well as ATP production in a dose dependent manner selectively in *ENO1* deleted cells (Supplemental Figure S6 and S7). Interestingly, while supplementation of 1 mM pyruvate attenuated the POMHEX-induced depression of OCR and ATP levels in both *ENO1* deleted as well as *ENO1* rescued cells, pyruvate only modestly rescued suppression of ECAR (Supplemental Figure S6, S7, S8). These results strongly suggest that pyruvate supplementation does not stimulate glycolysis through a shift in redox balance and that depletion of TCA cycle intermediates and bioenergetic collapse are the proximal consequences of POMHEX toxicity.

Interestingly, we also observed that lactate supplementation does not rescue POMHEX toxicity in gliomas (Supplementary Figure S4, S5 and S8). In a head to head comparison with pyruvate, we found that lactate cannot rescue enolase inhibition induced cell killing in both pyruvate-free DMEM as well as physiological Plasmax^TM^ medium (Supplementary Figure S4 and S8). Additionally, we also found that lactate is categorically unable to rescue POMHEX treatment induced depletion of ATP (Supplementary Figure S8). This strongly indicates that lactate is a poor mitochondrial substrate compared to pyruvate.

### Synergy of the enolase inhibitor POMHEX and glutaminase inhibitor CB-839 in vitro

**Fig. 3:**
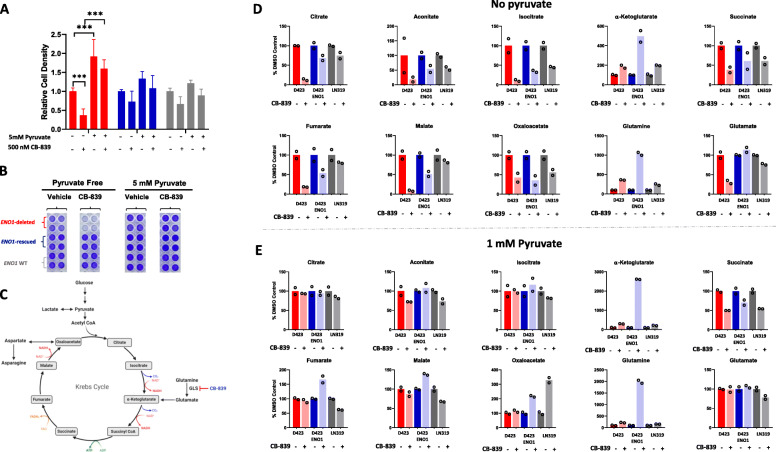
Media pyruvate availability modulates sensitivity to glutaminase inhibitor. **a-b** Cells were treated with 500 nM CB-839 in either pyruvate free DMEM or DMEM supplemented with 5 mM pyruvate for 5 days. Crystal violet staining was performed to determine the effect of CB-839 on cell growth. Cell density is expressed relative to vehicle control in pyruvate free medium. *ENO1* deleted *N*=16; *ENO1-*rescued *N*=16 and *ENO1* WT *N*=16; mean and +/− S.D. are shown. Where indicated, Asterisks represent statistical significance (*p*<0.0001) determined by two-way ANOVA and Tukey’s post hoc analysis. **c** Metabolic map representing the intermediates in the central carbon metabolic pathways. CB-839 targets glutaminase, the enzyme that converts glutamine to glutamate, and impedes glutamate anaplerosis to the TCA cycle. **d**, **e** Metabolomics analysis reveal TCA cycle intermediate depletion as a major consequence of CB-839 treatment. Cells were treated with 500 nM CB-839 in pyruvate free or regular DMEM (1 mM pyruvate) for 72 h and metabolites were extracted in 80% cold methanol and metabolite abundance was determined by MS. Representative TCA cycle metabolites that are altered by CB-839 treatment in pyruvate free **d** and pyruvate replete DMEM **e**

Cancer cells consume disproportionate amounts of the nonessential amino acid glutamine to support myriad biosynthetic reactions in the cells (Supplemental Figure S9C). To test whether *ENO1*-deleted cells exhibit glutamine auxotrophy, we grew *ENO1*-deleted, *ENO1*-rescued, and *ENO1*-wild type cells in glutamine-deficient and glutamine-supplemented medium. It is important to note that the glutamine-deficient DMEM was not exogenously supplemented with glutamine, but it was not devoid of glutamine since it contained FBS-derived glutamine. We observed that regardless of *ENO1* status, glutamine was essential for glioma cell growth as cells grown in glutamine-deficient medium displayed substantially delayed growth, confirming previous findings that cancer cells are “addicted” to glutamine in vitro (Supplemental Figure S9A-B) [11, 24]. Interestingly, in glutamine-deficient condition, treatment with the clinical-grade glutaminase inhibitor CB-839 instead of exacerbating cell death, to some extent restored cell proliferation (Supplemental Figure S9A-B). We reasoned that the small amount of FBS-derived glutamine that the cells are exposed to is quickly catabolized, rendering glutamine limiting for protein and nucleotide synthesis. CB-839 treatment can partially promote cell proliferation via glutamine accumulation upstream of the glutaminase reaction, which may be preserved for protein and nucleotide synthesis in the cells. This points to a dynamic role of the amino acid glutamine in cancer cells where glutamine-mediated non-anaplerotic reactions may be enough to support cancer cell survival and growth when exogenous supply of glutamine is limiting (Supplemental Figure S9A-C). Considering that glutamine is a highly abundant amino acid in plasma and those cells in vivo are constantly exposed to high amounts of glutamine, we were interested to explore how cancer cells depend on glutamine to support TCA cycle reactions. Thus, we tested whether abrogation of a step in the glutamine metabolism that is directly relevant to TCA cycle anaplerosis—i.e., glutaminase-mediated glutamine-to-glutamate conversion—could be modulated by addition of pyruvate to the medium (Fig. 3 a-b and Supplemental Figure S9C). To this end, we tested the efficacy of CB-839 under pyruvate-free condition and found that CB-839 significantly impaired the growth of *ENO1*-deleted cells (Fig. 3 a-b; Supplemental Figure S10). Additionally, the toxicity of CB-839 was reversed upon exogenous supplementation of high-dose anaplerotic substrates such as pyruvate and methyl-pyruvate, dimethyl 2-oxoglutarate and oxaloacetate, indicating that the efficacy of CB-839 can be modulated by the availability of anaplerotic substrates other than pyruvate (Fig. 3 a-b, and Supplemental Figure S10A-D). These findings further support the idea that the impairment of *ENO1*-deleted glioma cell growth upon abrogation of glutamine metabolism by CB-839 is indeed due to defects in TCA cycle anaplerosis. Interestingly, the addition of lactate did not rescue the toxicity of CB-839, reinforcing that under our current experimental conditions, gliomas do not prefer lactate as an anaplerotic substrate (Supplemental Figure S10C).

To mechanistically validate this, we performed metabolomic profiling of cells treated with the glutaminase inhibitor CB-839 in pyruvate-free and pyruvate-supplemented medium. We found that treatment with CB-839 in both pyruvate-free and pyruvate-supplemented conditions caused modest accumulation of glutamine (Fig. 3 d and e). CB-839 treatment also diminished glutamate levels in pyruvate-free conditions, although only in *ENO1*-deleted cells (Fig. 3 d). However, in pyruvate-supplemented conditions, CB-839 treatment did not diminish glutamate levels in any of the tested cell lines (Fig. 3 e). Additionally, under pyruvate-free conditions, CB-839 treatment caused a dramatic depletion of TCA cycle intermediates such as citrate, succinate, fumarate, malate, and oxaloacetate (Fig. 3 d). However, in pyruvate-supplemented conditions, the reduction in the levels of TCA cycle intermediates was not dramatic, suggesting that pyruvate-mediated anaplerosis attenuated the effect of CB-839 (Fig. 3 e). Our findings are consistent with recent studies showing that pyruvate-mediated anaplerosis is a major mechanism of resistance to CB-839 in in vitro tumor models of triple negative breast cancer [25].

**Fig. 4:**
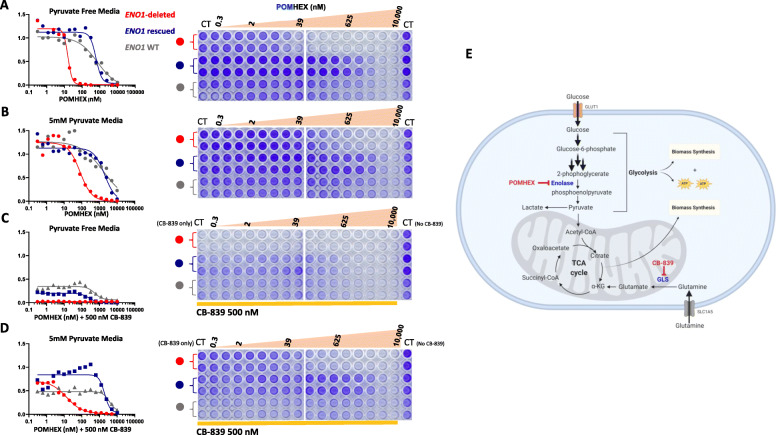
Synergistic anti-neoplastic effect of POMHEX and CB-839 in pyruvate free condition. **a-d** *ENO1* homozygously deleted (D423; red, *N*=2), *ENO1* isogenically rescued (D423 ENO1; blue, *N*=2) and *ENO1* WT (LN319; grey, *N*=2) cells were seeded in 96 well plates. After 24 h, the cells were treated with serial dilutions of POMHEX alone **a** and **b** or in combination with a fixed 500 nM CB-839 in pyruvate-free and pyruvate-supplemented medium (Columns 1-2 vehicle control; 3-10 serial dilutions of POMHEX and constant 500 nM CB-839; 11-12 constant 500 nM CB-839) **c** and **d**. The cells were grown in pyruvate free **a** and **c** or 5 mM pyruvate supplemented medium **b** and **d**. Following 5 days of drug treatment, cells were fixed and crystal violet staining was performed to determine cell density in response to the drug treatment. Data are expressed relative to the untreated control. Note the substantial synergy between POMHEX and CB-839 which is accentuated in pyruvate-free condition **c** and partially reversed by pyruvate supplementation **d**. **e** Schematic showing the inhibition of glycolysis by POMHEX at the enolase step, and inhibition of glutaminolysis by CB-839, both converging to impede TCA cycle anaplerosis, at different steps of the cycle

Given the interplay of pyruvate and glutamine to sustain the TCA cycle, we reasoned that these two arms of anaplerosis are redundant and together present a targetable metabolic liability in *ENO1*-deleted glioma cells. More specifically, due to the pronounced depletion of TCA cycle metabolites caused by POMHEX and CB-839 treatment individually, we hypothesized that impairing carbon atom restoration in the TCA cycle through these two pathways could synergize to specifically kill *ENO1*-deleted cells. Since CB-839 treatment alone showed a dramatic effect on cell viability in pyruvate-free conditions, we tested whether combined inhibition of glycolysis by POMHEX and glutaminolysis by CB-839 exacerbated toxicity specifically in *ENO1*-deleted cells. We found that *ENO1*-deleted cells were indeed dramatically sensitive to combined inhibition of glycolysis and glutaminolysis in pyruvate-free conditions (Fig. 4 a and c). Interestingly, this effect was partially rescued by addition of pyruvate (Fig. 4 d). These findings reinforce that glycolysis and glutaminolysis can be targeted together for the synergistic killing of *ENO1*-deleted cells by exacerbating the impairment of TCA cycle anaplerosis (Fig. 4 e).

### Co-treatment with HEX and CB-839 attenuates *ENO1*-deleted tumor growth but does not cause frank tumor regression

**Fig. 5:**
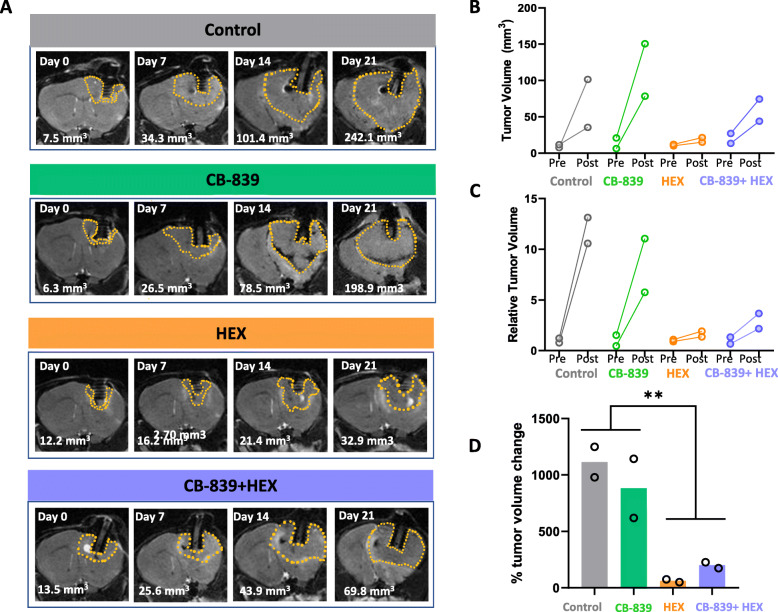
CB-839 and HEX combination attenuates intracranial tumor growth but does not cause a frank tumor regression. *ENO1-*deleted glioma cells (D423) were implanted intracranially in immunocompromised nude mice and tumor growth was monitored weekly by T2 MRI. Tumors are MRI detectable (indicated by dashed yellow outlines) 20-30 days after tumor implantation. 3D slicer was used to view the DICOM files and measure tumor volumes. **a** Representative MRI images to indicate weekly changes in tumor volume across different treatment groups, control (*N*=2), CB-839 treated (200 mpk BID orally; *N*=2), HEX treated (300 mpk SC; *N*=2), CB-839+HEX (200 mpk CB-839 BID orally, and 300 mpk HEX SC; *N*=2). **b** Pre- and post-treatment comparison of absolute and relative tumor volumes across different treatment groups. **c** Percent change in tumor volume after 2 weeks of drug treatment. Asterisks indicate statistical significance *p*<0.05 achieved by two-way ANOVA and Tukey’s post hoc analysis. Note that only the effect of HEX is significant

We next asked whether the synergistic effect observed in vitro from the combined inhibition of glycolysis and glutaminolysis would also take place in an in vivo tumor model. Since an intracranial orthotopic tumor would be the most faithful model for human glioma, we established *ENO1-*deleted intracranial tumors in immunocompromised *Foxn1*^*nu/nu*^ nude mice. Note that for our in vivo experiments, we used HEX instead of POMHEX to inhibit enolase. While POMHEX is superior to HEX in cell culture and is more potent (anti-tumor activity) on a molar basis in mice in vivo, the efficacy of POMHEX in mice is limited by its rapid degradation to the monoester form in mouse plasma (*t*_1/2_ <1 min) [9]. Thus, POMHEX requires administration through intravenous tail vein injections, which are difficult to carry out daily. At the same time, we wished to observe the interactions of enolase inhibition and glutaminase inhibition, which would be easier with a less potent enolase inhibitor. Nonetheless, despite HEX’s modest permeability across cell membranes, consistent with our previous data, we observed strong inhibition of intracranial tumor growth in mice bearing *ENO1*-deleted intracranial gliomas [9]. In contrast, following 3 weeks of drug administration, MRI-based tumor measurements indicated that the antineoplastic effect of CB-839 as a single agent was negligible (Fig. 5 a-d). Based on our in vitro data, we expected that treatment with both CB-839 and HEX would dramatically potentiate anaplerotic nutrient stress and induce synergistic attenuation of intracranial tumor growth. However, there was no hint that the combination treatment was better than HEX alone. Most likely, the antineoplastic effect we observed with the combination of HEX and CB-839 treatment was induced primarily by HEX. This drastic discrepancy in the efficacy of CB-839 alone and in combination with HEX in in vitro and in vivo settings could be due to CB-839’s poor blood-brain barrier (BBB) penetration, which has been reported previously [12].

**Fig. 6:**
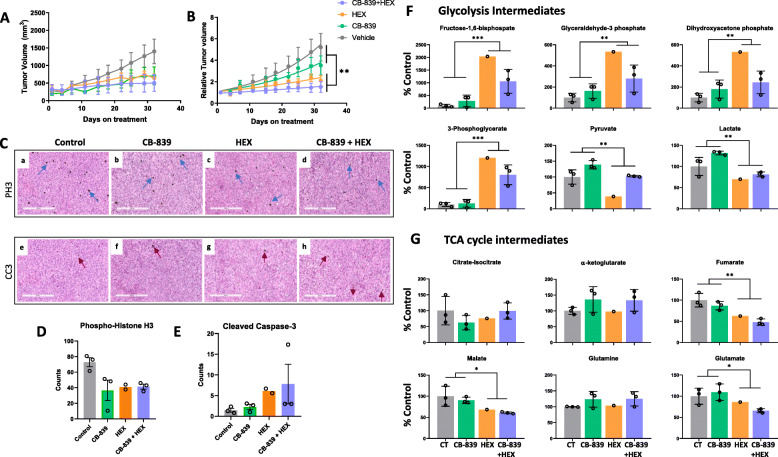
Glutaminase inhibition does not show anti-tumor activity against *ENO1*-deleted subcutaneous tumors. *ENO1-*deleted glioma cells (D423) were implanted subcutaneously in immunocompromised nude mice and once the tumors reached ~200 mm3, mice were randomly assigned into different treatment groups. Vehicle control (*N*=3), CB-839 (200 mpk BID orally; *N*=3), HEX (300 mpk SC; *N*=3), CB-839+HEX (200 mpk CB-839 BID orally, and 300 mpk HEX SC; *N*=3). **a-b** Tumor volume changes in response to the drug treatment was determined by measuring tumors three times a week using Vernier’s calipers. **a** Absolute (mm^3^) and **b** relative tumor growth curves during 1-month treatment course. Following the completion of treatment course, animals were sacrificed, and the tumors were dissected and fixed in formalin for histopathological analyses or frozen in liquid nitrogen for metabolomic analyses. **c** IHC staining for cell proliferation marker (phospho-histone H3, PH3; black stain, blue arrows) and marker of apoptosis (cleaved caspase-3, CC3; black stain, red arrows) in tissue sections of control, CB-839, HEX, and CB-839-HEX treated tumors. Size bar, 300 μm. **d-e** Counts of PH3 and CC3 positive cells per 100× section are shown. **f-g** Metabolomic analysis of frozen tumors show key differences in metabolites upstream and downstream of enolase reaction in HEX-treated tumors. Tumors were extracted approximately 4-6 h after the final dose. Representative glycolytic intermediates (top panel, **f**) and TCA cycle intermediates (bottom panel, **g**) altered in response to drug treatments. Metabolites are expressed relative to the vehicle control. (control, *N*=3; CB-839, *N*=3; HEX, *N*=1; and CB-839-HEX, *N*=3; mean and +/− S.D. where relevant with individual data points are shown). Where indicated, asterisks represent statistical significance (*p*<0.05) achieved by two-way ANOVA and Tukey’s post hoc analysis. (Metabolomics data represented in this panel were obtained using metabolomics core at BIDMC and included *N*=1 HEX-treated tumor. Remaining HEX-treated tumors from this experiment were used for metabolomics with the Metabolon Inc. platform. See Supplemental Figure S11)

In order to rule out the limitations imposed by the BBB in drug delivery to the tumors, we tested the combination of CB-839 and HEX in a subcutaneous tumor model. We subcutaneously injected *ENO1*-deleted D423 cells into immunocompromised nude *Foxn1*^*nu/nu*^ mice. After the tumors reached an approximate size of 150 mm^3^, we treated the mice with CB-839 and HEX as single agents and in combination. On its own, CB-839 only minimally diminished subcutaneous tumor growth, whereas consistent with our observations of intracranial tumor growth, HEX on its own had a strong antineoplastic effect (Fig. 6 a and b). Interestingly, although in the intracranial tumor model, the efficacy of the combination of CB-839 and HEX was almost indistinguishable from that of the single-drug treatments, especially HEX, in subcutaneous models, the combination treatment was modestly more efficacious, indicating a possible additive effect of the two drugs (Fig. 6 a and b). Immunohistochemical staining for phosphor-histone H3 (PH3) revealed that compared to the control group, there were significantly fewer actively dividing cells in CB-839, HEX, and CB-839 + HEX-treated tumors (Fig. 6 c, upper panel, and Fig. 6 d). However, cleaved-caspase 3 (CC3) signal corroborated the pattern observed on the tumor growth curve, where CB-839 treatment induced only minimal apoptosis, while HEX and combination of CB-839 and HEX led to considerable tumor apoptosis (Fig. 6 c, lower panel, and Fig. 6 e).

Next, to discern the effect of the drugs on the metabolite profiles, we performed metabolomic analyses of frozen sections of the subcutaneous tumors. Consistent with our in vitro metabolomic data, we found that treatment with HEX, both as a single agent and in combination with CB-839, led to a dramatic accumulation of glycolytic intermediates upstream of enolase while significantly diminishing downstream metabolites such as pyruvate and lactate (Fig. 6 f and Supplemental Figure S11). CB-839 treatment, however, despite showing modest target engagement as evidenced by accumulation of glutamine, did not cause notable reductions in TCA cycle metabolites (Fig. 6 g). HEX treatment on its own elicited notable reductions in TCA cycle metabolites such as fumarate and malate, but the addition of CB-839 did not significantly enhance this effect of HEX (Fig. 6 g). Moreover, the extent of TCA cycle disruption corresponded to the extent of tumor growth inhibition in each treatment group. Thus, we confirmed that even when CB-839’s BBB permeability was not a limiting factor, the synergy between HEX and CB-839 that we observed in in vitro experiments could not be recapitulated in in vivo subcutaneous tumor models and, at best, the combination treatment exhibited an additive interaction. This suggests that *ENO1*-deleted glioma cells minimally depend on glutamine as a fuel to support anaplerosis in vivo.

## Discussion

Inhibition of central carbon metabolism has long been an aspirational target for the treatment of cancer. As the focal point of central carbon metabolism, the TCA cycle needs to be continuously replenished with carbon atoms that are drained for the synthesis of biosynthetic building blocks and catabolic reactions feeding the mitochondrial electron transport chain [13, 14]. It is thought that the main sources of anaplerotic substrates for cancer cells are glutamate derived from glutamine and pyruvate derived from glucose [14]. Studies have demonstrated that a compromise in the pyruvate supply to the TCA cycle renders glutamine oxidation essential for TCA cycle anaplerosis, establishing a synthetic lethal interaction that could be exploited for cancer therapy [26, 27]. The recent development of high-affinity inhibitors of glutaminase such as CB-839 has led to a flurry of studies on the role of glutaminolysis in sustaining cancer cell growth. Similarly, our lab has recently synthesized and systematically validated high-affinity inhibitors of the glycolytic enzyme enolase and has drawn attention to the existence of subsets of cancers with homozygous deletions of *ENO1*, which are deficient in overall enolase activity and susceptible to inhibitors of oxidative phosphorylation by virtue of this glycolytic deficiency [9, 28]

In this study, we brought together our novel inhibitors of enolase, HEX, and POMHEX, as well as the glutaminase inhibitor CB-839, to investigate the redundancies between glycolysis and glutaminolysis for supporting tumor growth and viability in vitro and in vivo in *ENO1*-deleted gliomas. First, we performed a systematic metabolomic analysis to determine the effects of the enolase inhibitor prodrug, POMHEX, on cultured glioma cells varying in *ENO1*-deletion status. We found that even in DMEM with supraphysiological levels of glucose (25 mM, fivefold higher than physiological), pyruvate (1 mM, 30-fold higher than physiological), and glutamine (4 mM, eightfold higher than physiological) [29], the inhibition of enolase in multiple glioma cell lines resulted in significant depletion of TCA cycle metabolites (Fig. 1 e, Supplemental Figure S1 and S2), underscoring the critical role of glucose-derived pyruvate in supporting anaplerosis. The degree of TCA cycle metabolite depletion correlated with the cell lines’ *ENO1* status and their sensitivity to enolase inhibitors (Fig. 1 a-c). Given the close correlation between TCA cycle metabolite depletion (inhibition of anaplerosis) and sensitivity to enolase inhibitors (Fig. 1 a-e), we determined the effect of experimental manipulation of anaplerotic nutrients, first by supplementing with exogenous anaplerotic substrates and second by inhibition of glutaminolysis with CB-839. We found that omission of exogenous pyruvate in DMEM significantly sensitized all glioma cell lines to the enolase inhibitor POMHEX, irrespective of *ENO1* status (Fig. 2 b, Supplemental Figure S3). Supplementation with physiological levels of pyruvate (100 μM) provided some rescue, with the effect peaking at 1 mM (data not shown) and no further benefit at 5 mM. Similar rescue was obtained with methyl-pyruvate, a cell-permeable pro-metabolite, as well as with oxaloacetate and oxovalerate (Fig. 2 b and Supplemental Figure S4). These observations draw a metabolic portrait that establishes pyruvate as a major contributor to TCA cycle anaplerosis. However, it is important to note that abrogation of anaplerotic pyruvate by the enolase inhibitor is not the only mechanism of toxicity, as exogenous supplementation of pyruvate could not completely rescue POMHEX toxicity. These points to other non-anaplerotic pathways that are altered as a result of glycolysis inhibition and may contribute to cell killing.

The promiscuous nature of pyruvate means that it has the potential to provide POMHEX toxicity relief through alternative mechanisms such as through a shift in the NAD+/NADH redox balance and stimulation of glycolysis. Our results conclusively evidence that POMHEX treatment renders *ENO1* deleted gliomas substrate limited for mitochondrial respiration, and supplementation of pyruvate mitigates POMHEX toxicity by restoring mitochondrial respiration and ATP production and only minimally by NAD+/NADH redox shift and stimulation of glycolysis (Supplemental Figure S5, S6, S7). In this context, pyruvate can serve as a mitochondrial substrate through both the pyruvate carboxylase (PC) and pyruvate dehydrogenase reactions (PDH). Through, U-13C pyruvate labeling study, we show that pyruvate can enter the TCA cycle through both PC and PDH reactions (Supplementary Figure S12). In the context of enolase inhibition, pyruvate flux through both PC and PDH reactions is increased, reinforcing that POMHEX treatment renders *ENO1* deleted cells limited for mitochondrial substrates (anaplerosis and respiration) and that pyruvate can rescue POMHEX toxicity through both mechanisms. However, we also found that direct mitochondrial respiratory substrates that produce acetyl-CoA, such as acetate and fatty acids, are not as effective as pyruvate in providing POMHEX toxicity relief (Supplemental Figure S4E and H). Similarly, we also found that the toxicity of POMHEX is exacerbated in low CO_2_ conditions, and the effect ameliorated partially when CO_2_ exposure is increased, which is consistent with the anaplerotic requirement of CO_2_ for pyruvate carboxylase reaction (Supplemental Figure S13). Overall, these data emphasize that in POMHEX-treated conditions, the classical anaplerotic PC reaction is deficient, and pyruvate supplementation rescues POMHEX toxicity through both PC and PDH mechanisms.

Finally, we investigated the effect of glutaminolysis inhibition, given the importance of this pathway for anaplerosis. We found that glioma cells with *ENO1* homozygous deletions are selectively sensitive to CB-839; however, this was entirely reversed by exogenous pyruvate supplementation (Fig. 3 a-b). Metabolomic profiling of CB-839 treated *ENO1*-deleted, isogenic rescued, and intact glioma cells underscored the importance of exogenous pyruvate, as CB-839 treatment resulted in significant TCA cycle metabolite depletion only in the absence of pyruvate (Fig. 3 d-e). Strikingly, the selective toxicity of CB-839 was also rescued by the same anaplerotic nutrients as that of enolase inhibition (Fig. 3 a-b and Supplemental Figure S4 and S10). Similarly, the combination of enolase and glutaminase inhibitors in vitro proved very interesting: the two treatments clearly agonized each other, with their effects being at least additive, and—depending on the drug dose, treatment length, and nutrient composition of the media—synergistic.

With these encouraging results in hand, we tested the antitumor and metabolic effects of enolase and glutaminase inhibitors in vivo. In an intracranial tumor orthotopic model, we saw no effects with CB-839 alone—and no additive effects with the enolase inhibitor HEX—against tumors with *ENO1* homozygous deletions (Fig. 5 a-d). The lack of an antitumor effect of CB-839 could be ascribed to its inability to cross the BBB, though we have noted that D423 tumors do in fact have a breached BBB [9]. We therefore repeated these experiments with the same cell line but implanted the tumor subcutaneously. Unexpectedly, we found that the synergy was not conserved in vivo, as HEX’s antineoplastic efficacy was comparable to the efficacy achieved by combining HEX and CB-839, indicating that any antineoplastic effect obtained by combining HEX and CB-839 could largely be attributed to the impairment of glycolysis by HEX (Fig. 6 a and b). Thus, we argue that bioenergetic collapse, together with reduced pyruvate flux to the TCA cycle, accounts for the dramatic antineoplastic efficacy of HEX against *ENO1*-deleted gliomas and that glutamine oxidation may not be obligatory for tumor sustenance in vivo.

Our study addresses one of the outstanding controversies in the field: What is the predominant source of carbon atoms for the TCA cycle in cancer cells? Although multiple studies have offered unquestionable evidence that excessive glucose flux and oxidation are near-universal characteristics of cancer cells, the net contribution of different carbon sources such as glucose, glutamine, lactate, and acetate is still an unresolved question [30, 31]. Isotope-based labeling studies have attempted to delineate the source of carbon atoms to the TCA cycle in both in vitro and in vivo model systems. For example, some studies have reported that certain tissues and tumors prefer lactate over other substrates as a source of energy [32–34], while other studies have pinpointed glucose rather than lactate as the predominant contributor to the TCA cycle in most tissues [31, 35]. Our data do not support that lactate or acetate can be utilized by cells instead of pyruvate to support TCA cycle anaplerosis (Supplemental Figure S4G-H, S5, S8). This is probably because lactate isotope exchange at equilibrium has been reported to overestimate actual lactate flux and net lactate contribution to the TCA cycle [31, 35]. Additionally, in an in vivo setting, the stroma-tumor metabolic coupling may allow lactate extruded by highly glycolytic tumor cells to be used as fuel by stromal cells. In return, stromal cells may reciprocally support the metabolic demands of tumor cells by sharing pyruvate with the tumor cells [36, 37]. Similarly, while other groups have shown that tumor cells can directly use acetate as a source of carbon to replenish TCA cycle intermediates, we did not see any significant reversal of toxicity of glycolysis inhibition with acetate supplementation [38]. There are several possible explanations for the differences between acetate and pyruvate. The most obvious is that the transition from pyruvate into the TCA cycle is rapid, energetically favorable, and carried out by high-abundance enzymes, pyruvate dehydrogenase as well as pyruvate carboxylase. The incorporation of acetate into the TCA cycle requires ATP hydrolysis and is predominantly cytosolic. We speculate that anaplerosis by this pathway is simply too slow to compensate for the diminished pyruvate production from inhibited glycolysis.

Furthermore, we observed that the anaplerotic and non-anaplerotic functions of glutamine are dynamic and are dictated by the availability of exogenous glutamine. More specifically, when exogenous glutamine was limiting, we found that cell survival, regardless of *ENO1* status, was severely compromised (Supplemental Figure S9). However, addition of CB-839, which under glutamine replete conditions suppressed tumor growth in vitro, but in glutamine-free conditions, led to partial rescue of tumor growth. This indicates that anaplerotic functions of glutamine are supported only when glutamine levels are abundant, and CB-839 treatment can indeed impair tumor growth. However, when exogenous glutamine levels are limiting as would be the case in poorly perfused hypoxic regions of a tumor, CB-839 treatment may in fact promote tumor growth via accumulation of endogenous glutamine which is now available for nucleotide and amino acid synthesis. The delineation of anaplerotic and non-anaplerotic roles of glutamine and the contexts in which they may be predominant, is therefore critical especially given that CB-839 is currently being investigated in phase II clinical trials for multiple malignancies.

Yet, despite a promising observation with CB-839 in vitro (Fig. 3 a-b), we found that in vivo, glutamine may not be a prominent anaplerotic substrate and that glucose-derived pyruvate predominantly drives anaplerosis, at least in *ENO1*-deleted gliomas (Figs. 5 and 6). Despite its abundance in cell culture medium (4 mM in DMEM) and in human plasma (0.5 mM), the generalizable extent to which cancer cell lines in culture require glutamine for their survival remains controversial [29, 39–42]. Additionally, the switch of cancer cells from auxotrophy in vitro to independence from glutamine catabolism in vivo, as reported previously and reinforced in our study, compounds this question [43]. Similarly, if tissue culture media are supplemented with non-physiological levels of nutrients including glutamine, multiple confounding parameters are introduced that do not resemble an in vivo metabolite profile. For example, Muir et al. [44] reported that high levels of cystine in tissue culture medium render cells sensitive to glutaminase inhibition through cystine’s role in glutamate transport via the cystine/glutamate antiporter xCT/SLC7A11. Indeed, we also observed that the toxicity of CB-839 in *ENO1*-deleted cells is significantly attenuated in physiological Plasmax^TM^ medium compared to DMEM medium (Supplemental Figure S14), which implies that in vivo tumor microenvironment may not be conducive to glutamine addiction, at least for ENO1-deleted gliomas. Glutamine catabolism independence has also been reported in other in vivo tumor models [39]. In the context of glioblastoma, some studies have maintained that pyruvate-derived carbons dominate those coming from glutamine in the TCA cycle [39, 40], while another recent study showed that the origin of the glioblastoma cells influences their preference of anaplerotic substrate [45]. Overall, dependence on glutamine oxidation appears to be heterogenous, and dictated by underlying genetics [43, 46], tissue origin [46], and tumor microenvironment [44, 47], and glutamine auxotrophy may not be a universal characteristic of cancer cells in vivo.

## Conclusions

Our study emphasizes the critical role of glycolysis-derived pyruvate for replenishing the TCA cycle. We also found that under nutrient conditions that approximate the in vivo metabolic environment, glutaminase inhibition has only modest effects even in ENO1-deleted glioma cells, which are quite sensitive in “normal” media. Our results emphasize the importance of selecting culture media that most closely reflect the in vivo nutrient profile, to more accurately predict the efficacy of metabolism-targeting drugs. At the same time, our results also suggest that tumor microenvironment depleted of anaplerotic nutrients, can potentially sensitize cancer cells to inhibitors of central carbon metabolism. Such a situation might arise in response to angiogenesis inhibition, where tumors are deprived of plasma-borne nutrient flow. Future work will be required to test this hypothesis.

## Supplementary Information

**Additional file 1: Supplemental Figure S1.** POMHEX treatment results in accumulation of glycolytic metabolites upstream of enolase. **Supplemental Figure S2.** POMHEX treatment leads to an overall reduction in TCA cycle metabolites. **Supplemental Figure S3.** Pyruvate rescues POMHEX toxicity in glioma cells. **Supplemental Figure S4.** Rescue of POMHEX toxicity by anapleroticsubstrates. **Supplemental Figure S5.** Exogenously supplemented, supraphysiological levels of anapleroticsubstrates rescue POMHEX toxicity even in physiological PlasmaxTMmedium. **Supplemental Figure S6.** POMHEX treated cells are substrate limited for mitochondrial respiration which is attenuated by exogenous pyruvate. **Supplemental Figure S7.** Enolase inhibition induces bioenergetics stress preceding cell killing, which is rescued by exogenous pyruvate. **Supplemental Figure S8.** Exogenous pyruvate but not lactate rescues ATP production inhibited by enolase inhibitor treatment*.* **Supplemental Figure S9.** Glioma cells exhibit glutamine auxotrophy*in vitro.* **Supplemental Figure S10.** CB-839 toxicity is exaggerated under pyruvate free conditions and reversed by the addition of anapleroticsubstrates. **Supplemental Figure S11.** Metabolomic analysis of HEX treated *ENO1*deleted tumors from two different metabolomic platforms confirm elevation of glycolytic metabolites upstream, and reduction in the TCA cycle metabolites, downstream of the enolase reaction. **Supplemental Figure S12.** Exogenously supplemented pyruvate contributes to TCA cycle through both pyruvate carboxylase and pyruvate dehydrogenase reactions. **Supplemental Figure S13.** CO2levels modulate POMHEX toxicity. **Supplemental Figure S14.** Sensitivity of glioma cells to CB-839 is attenuated in physiological PlasmaxTMmedium. **Supplemental Figure S15.** Orally administered CB-839 is detectable in mouse plasma 2 hours post drug administration. via LC-MS (ESI).

## Abbreviations

2-OG: 2-Dimethyloxoglutarate
BBB: Blood-brain barrier
BIDMC: Beth Israel Deaconess Medical Center
CCLC: Characterized Cell Line Core
IACUC: Institutional Animal Care and Use Committee
IC_50_: Half maximal inhibitory concentration
MRI: Magnetic resonance imaging
PBS: Phosphate-buffered saline
TCA: Tricarboxylic acid
ATP: Adenosine triphosphate
PH3: Phospho-histone H3
CC3: Cleaved caspase-3
AKB: α-Ketobutyric acid
OCR: Oxygen consumption rate
ECAR: Extracellular acidification rate
LDH: Lactate dehydrogenase
PC: Pyruvate carboxylase
PDH: Pyruvate dephydrogenase
