# Supplementary material for: Impaired anaplerosis is a major contributor to glycolysis inhibitor toxicity in glioma
Source: Cancer Metab. 2021 Jun 25;9:27. doi: 10.1186/s40170-021-00259-4 (PMC8228515; doi:10.1186/s40170-021-00259-4)
Supplement: Supplementary file 1 — Additional file 1: Supplemental Figure S1. POMHEX treatment results in accumulation of glycolytic metabolites upstream of enolase. Supplemental Figure S2. POMHEX treatment leads to an overall reduction in TCA cycle metabolites. Supplemental Figure S3. Pyruvate rescues POMHEX toxicity in glioma cells. Supplemental Figure S4. Rescue of POMHEX toxicity by anapleroticsubstrates. Supplemental Figure S5. Exogenously supplemented, supraphysiological levels of anapleroticsubstrates rescue POMHEX toxicity even in physiological PlasmaxTMmedium. Supplemental Figure S6. POMHEX treated cells are substrate limited for mitochondrial respiration which is attenuated by exogenous pyruvate. Supplemental Figure S7. Enolase inhibition induces bioenergetics stress preceding cell killing, which is rescued by exogenous pyruvate. Supplemental Figure S8. Exogenous pyruvate but not lactate rescues ATP production inhibited by enolase inhibitor treatment. Supplemental Figure S9. Glioma cells exhibit glutamine auxotrophyin vitro. Supplemental Figure S10. CB-839 toxicity is exaggerated under pyruvate free conditions and reversed by the addition of anapleroticsubstrates. Supplemental Figure S11. Metabolomic analysis of HEX treated ENO1deleted tumors from two different metabolomic platforms confirm elevation of glycolytic metabolites upstream, and reduction in the TCA cycle metabolites, downstream of the enolase reaction. Supplemental Figure S12. Exogenously supplemented pyruvate contributes to TCA cycle through both pyruvate carboxylase and pyruvate dehydrogenase reactions. Supplemental Figure S13. CO2levels modulate POMHEX toxicity. Supplemental Figure S14. Sensitivity of glioma cells to CB-839 is attenuated in physiological PlasmaxTMmedium. Supplemental Figure S15. Orally administered CB-839 is detectable in mouse plasma 2 hours post drug administration. via LC-MS (ESI). [file 40170_2021_259_MOESM1_ESM.pdf]

Supplementary Figures

Supplemental Figure S1. POMHEX treatment results in accumulation of glycolytic metabolites upstream of enolase

Supplemental Figure S2. POMHEX treatment leads to an overall reduction in TCA cycle metabolites.

Supplemental Figure S3. Pyruvate rescues POMHEX toxicity in glioma cells.

Supplemental Figure S4. Rescue of POMHEX toxicity by anaplerotic substrates.

Supplemental Figure S5. Exogenously supplemented, supraphysiological levels of anaplerotic substrates rescue POMHEX toxicity even in physiological Plasmax^{TM} medium.

Supplemental Figure S6. POMHEX treated cells are substrate limited for mitochondrial respiration which is attenuated by exogenous pyruvate.

Supplemental Figure S7. Enolase inhibition induces bioenergetics stress preceding cell killing, which is rescued by exogenous pyruvate.

Supplemental Figure S8. Exogenous pyruvate but not lactate rescues ATP production inhibited by enolase inhibitor treatment.

Supplemental Figure S9. Glioma cells exhibit glutamine auxotrophy *in vitro.*

Supplemental Figure S10. CB-839 toxicity is exaggerated under pyruvate free conditions and reversed by the addition of anaplerotic substrates.

Supplemental Figure S11. Metabolomic analysis of HEX treated *ENO1* deleted tumors from two different metabolomic platforms confirm elevation of glycolytic metabolites upstream, and reduction in the TCA cycle metabolites, downstream of the enolase reaction.

Supplemental Figure S12. Exogenously supplemented pyruvate contributes to TCA cycle through both pyruvate carboxylase and pyruvate dehydrogenase reactions.

Supplemental Figure S13. CO_2 levels modulate POMHEX toxicity.

Supplemental Figure S14. Sensitivity of glioma cells to CB-839 is attenuated in physiological Plasmax^{TM} medium.

Supplemental Figure S15. Orally administered CB-839 is detectable in mouse plasma 2 hours post drug administration. via LC-MS (ESI).

Figure S1

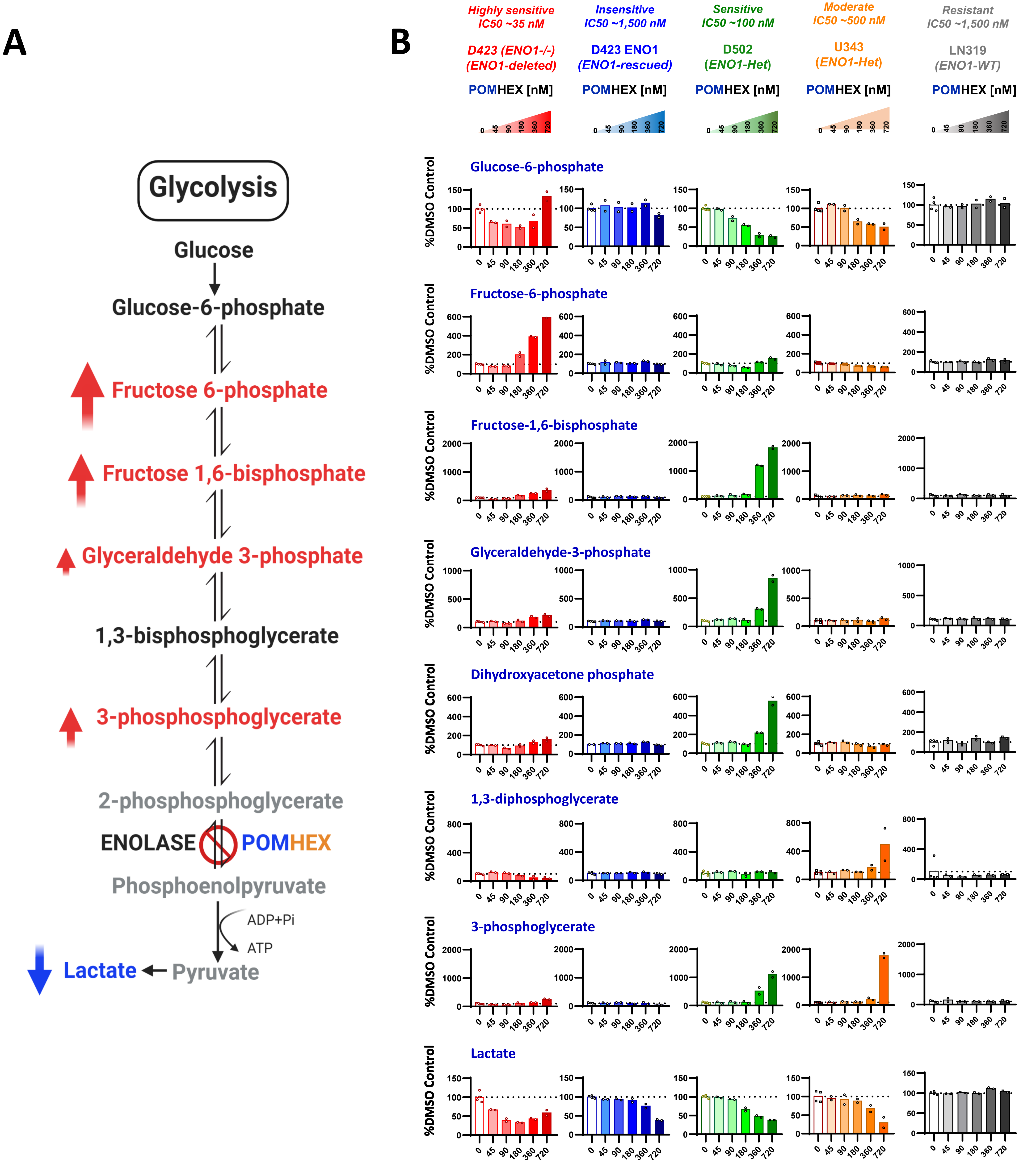

A
Glycolysis
Glucose
Glucose-6-phosphate
Fructose 6-phosphate
Fructose 1,6-bisphosphate
Glyceraldehyde 3-phosphate
1,3-bisphosphoglycerate
3-phosphophoglycerate
2-phosphophoglycerate
ENOLASE
POMHEX
Phosphoenolpyruvate
ADP+Pi
ATP
Lactate
Pyruvate
B
Highly sensitive IC50 ~35 nM
D423 (ENO1-/-) (ENO1-deleted)
Insensitive IC50 ~1,500 nM
D423 ENO1 (ENO1-rescued)
Sensitive IC50 ~100 nM
D502 (ENO1-Het)
Moderate IC50 ~500 nM
U343 (ENO1-Het)
Resistant IC50 ~1,500 nM
LN319 (ENO1-WT)
POMHEX [nM]
0 45 90 180 360 720
%DMSO Control
Glucose-6-phosphate
Fructose-6-phosphate
Fructose-1,6-bisphosphate
Glyceraldehyde-3-phosphate
Dihydroxyacetone phosphate
1,3-diphosphoglycerate
3-phosphoglycerate
Lactate

Supplemental Figure S1.**POMHEX treatment elevates the levels of glycolytic intermediates upstream of Enolase**. **A.** Schematic showing the glycolytic intermediates that are altered by POMHEX treatment. **B.** *ENO1* homozygously deleted (D423,red), *ENO1*-isogenic rescue (D423 *ENO1*, blue), *ENO1* heterozygous (D502, green; U423, orange) and *ENO1* wild type (LN319, grey) were treated with serial dilution of enolase inhibitor POMHEX for 72 hours and metabolomics analysis was performed on the extracted intracellular metabolites (N=2 biological replicates). The level of each metabolite is expressed relative to the DMSO control group. Treatment with POMHEX leads to an overall and significant accumulation of metabolites upstream of enolase reaction. Note that the degree of metabolite accumulation correlates with the sensitivity of each cell line to POMHEX (**Figure 2**).

Figure S2

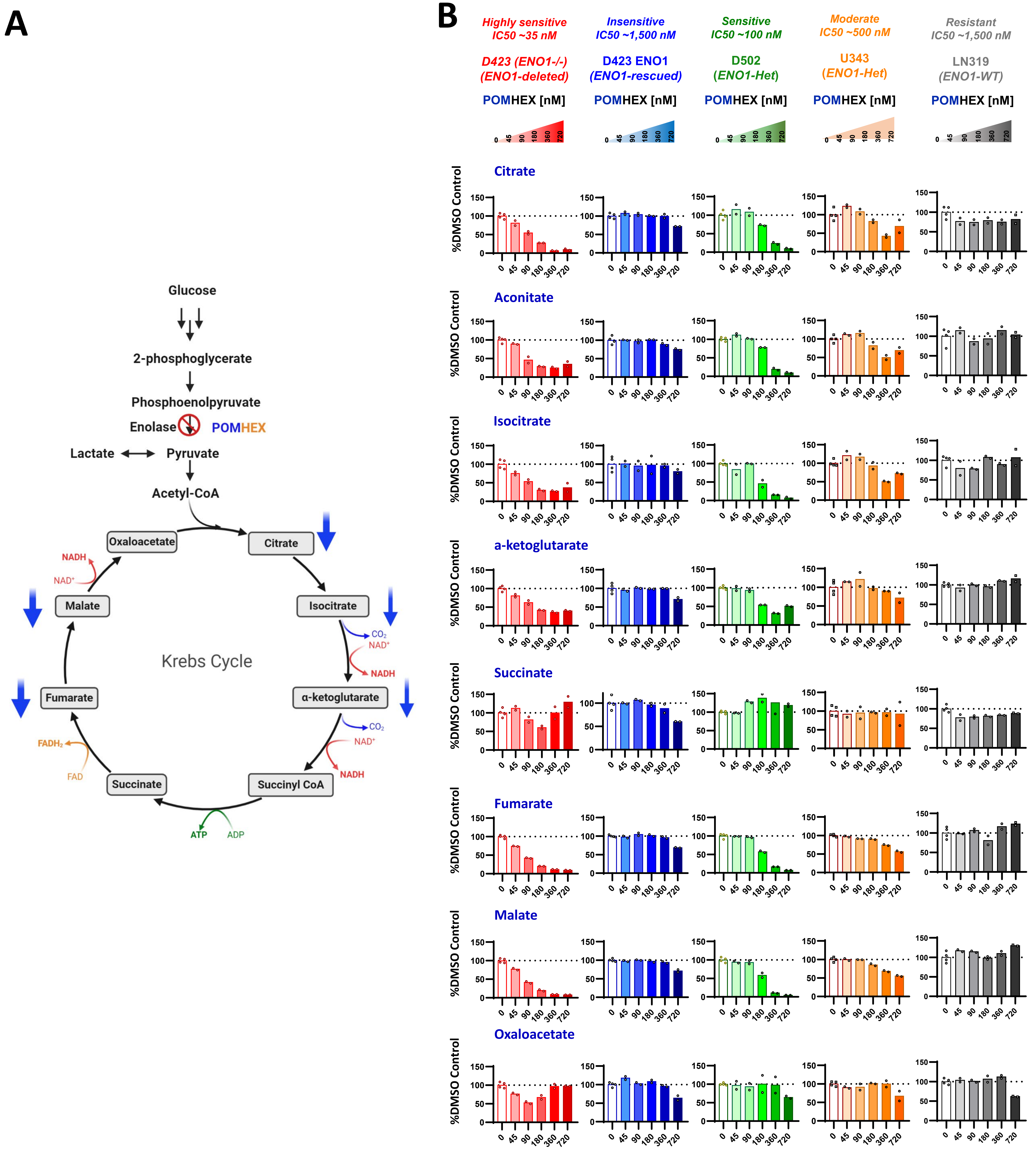
A
Glucose
2-phosphoglycerate
Phosphoenolpyruvate
Enolase
POMHEX
Lactate
Pyruvate
Acetyl-CoA
Oxaloacetate
Citrate
Isocitrate
α-ketoglutarate
Succinyl CoA
Succinate
Fumarate
Malate
Krebs Cycle
NADH
NAD+
CO2
FADH2
FAD
ATP
ADP
B
Highly sensitive IC50 ~35 nM
D423 (ENO1-/-) (ENO1-deleted)
Insensitive IC50 ~1,500 nM
D423 ENO1 (ENO1-rescued)
Sensitive IC50 ~100 nM
D502 (ENO1-Het)
Moderate IC50 ~500 nM
U343 (ENO1-Het)
Resistant IC50 ~1,500 nM
LN319 (ENO1-WT)
POMHEX [nM]
0 45 90 180 360 720
%DMSO Control
Citrate
Aconitate
Isocitrate
a-ketoglutarate
Succinate
Fumarate
Malate
Oxaloacetate

Supplemental Figure S2. **TCA cycle metabolites decrease in response to Enolase inhibitor POMHEX**. **A.** Schematic showing the contributions of pyruvate and glutamine at different steps of the TCA cycle. **B.** *ENO1* homozygously deleted (D423,red), *ENO1*-isogenic rescue (D423 *ENO1*, blue), *ENO1* heterozygous (D502, green; U343, orange) and *ENO1* wild type (LN319, grey) were treated with the enolase inhibitor POMHEX at the indicated doses for 72 hours and polar metabolites were extracted (N=2 biological replicates). Metabolites are expressed relative to the DMSO control. Metabolomics analysis show an overall decrease in the levels of TCA cycle metabolites downstream of the enolase reactions. The degree of disruption in the levels of TCA cycle intermediates correlates with the sensitivity of each cell line to the inhibitor (**Figure 2**).

Figure S3

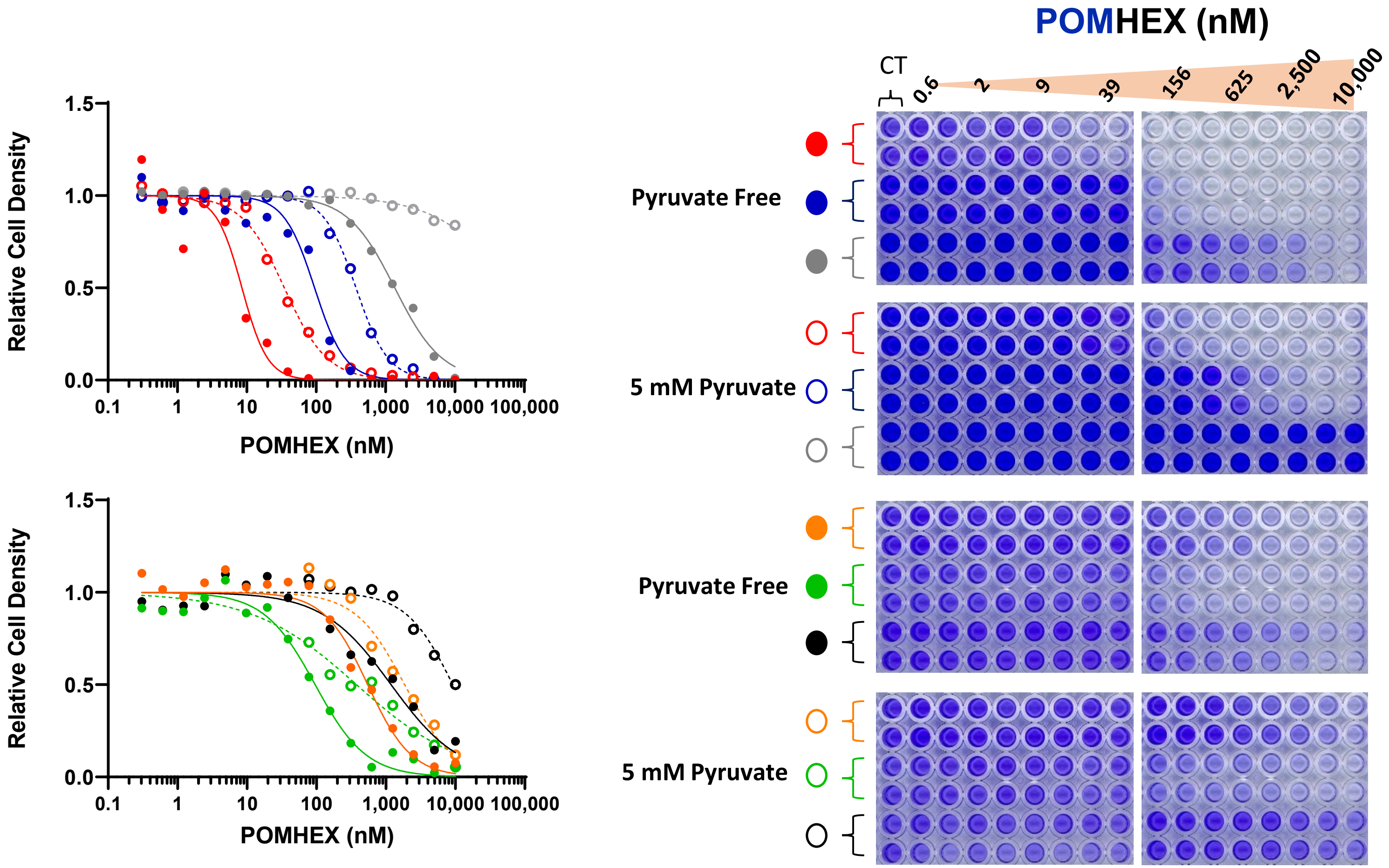

	No Pyruvate	5 mM Pyruvate	
	IC_{50} (nM)	IC_{50} (nM)	n
D423 (*ENO1*-deleted)	9	35	2
U343 (*ENO1*-Heterozygous)	547	1,863	2
D502 (*ENO1*-Heterozygous)	96	377	2
D423 (*ENO1*-rescued)	95	376	2
LN319 (*ENO1*-WT)	1,360	>10,000	2
Astrocytes (*ENO1*-WT)	1,187	9,198	2

Supplemental Figure S3. **Pyruvate rescues POMHEX toxicity in glioma cells**. **A.** *ENO1* homozygously deleted (D423; red, N=2), *ENO1*-isogenic rescue (D423 *ENO1;* blue, N=2), *ENO1* heterozygous (D502; green, N=2; U423; orange, N=2) and *ENO1* wild type (LN319; grey, N=2; and astrocytes, black; N=2). Cells were treated with varying concentrations of POMHEX in pyruvate free or 5mM pyruvate supplemented DMEM for 5 days. On day 5, cells were fixed in 10% formalin and stained with crystal violet to determine terminal cell density. Cell density is expressed relative to vehicle controls. **B**. Table indicating the IC_{50} values of POMHEX for each cell line in pyruvate free and 5mM pyruvate supplemented DMEM. Note that addition of pyruvate rescues POMHEX toxicity in all cell lines tested.

Figure S4

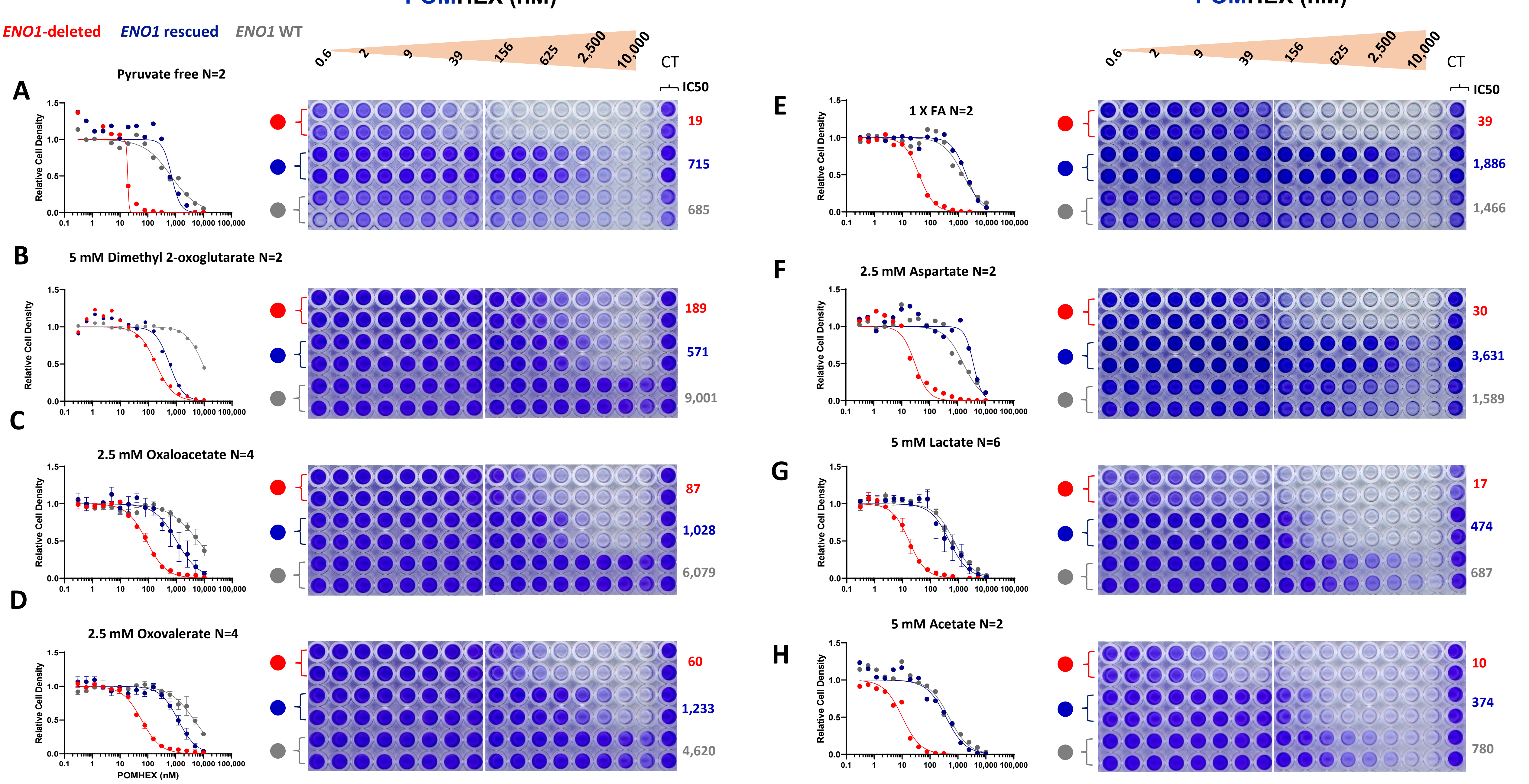

POMHEX (nM)
POMHEX (nM)
ENO1-deleted
ENO1 rescued
ENO1 WT
0.6
2
9
39
156
625
2,500
10,000
CT
IC50
A
Pyruvate free N=2
19
715
685
B
5 mM Dimethyl 2-oxoglutarate N=2
189
571
9,001
C
2.5 mM Oxaloacetate N=4
87
1,028
6,079
D
2.5 mM Oxovalerate N=4
60
1,233
4,620
E
1 X FA N=2
39
1,886
1,466
F
2.5 mM Aspartate N=2
30
3,631
1,589
G
5 mM Lactate N=6
17
474
687
H
5 mM Acetate N=2
10
374
780
Relative Cell Density
POMHEX (nM)
0.1
1
10
100
1,000
10,000
100,000
0.0
0.5
1.0
1.5

Supplemental Figure S4. **Rescue of POMHEX toxicity by anaplerotic substrates**. *ENO1* homozygously deleted (D423; red), *ENO1*-isogenically rescued (D423 *ENO1*; blue), and *ENO1*-WT (LN319; grey) cells were treated with serial dilutions of POMHEX at the indicated doses in pyruvate free DMEM (**A**, N=2) or DMEM exogenously supplemented with 5 mM dimethyl 2-oxoglutarate (**B**, N=2), 2.5 mM oxaloacetate (**C**, N=4) , 2.5 mM oxovalerate (**D**, N=4), 1 X Fatty acid (**E**, N=2) and 2.5 mM aspartate (**F**, N=2),5mM Lactate (**G**, N=6), 5mM acetate (**H**, N=4). Following 5-days of drug treatment, cell density was measured by crystal violet staining. Cell density is expressed relative to the DMSO control groups. Where indicated, error bars represent standard error of the mean. IC_{50} of POMHEX for the cell lines in each medium condition is indicated. Note that the anaplerotic substrate dimethyl 2-oxoglutarate provides the best rescue to POMHEX toxicity, while lactate as well as mitochondrial respiration substrates such as fatty acids and acetate provide minimal rescue to POMHEX toxicity.

Figure S5

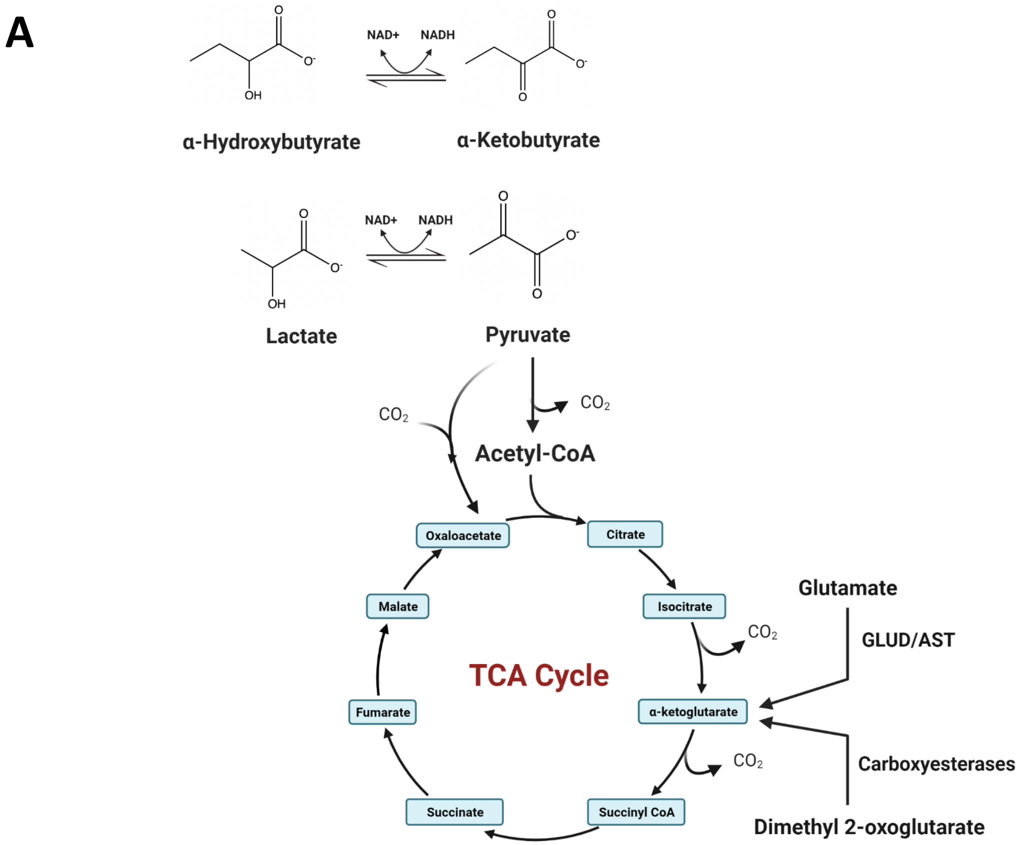

B

Cells	Plasmax IC_{50} (nM)	5 mM Pyruvate IC_{50} (nM)	5mM AKB IC_{50} (nM)	5 mM 2OG IC_{50} (nM)	5 mM Glutamate IC_{50} (nM)	5 mM Lactate IC_{50} (nM)
ENO1-deleted	67	313	116	331	40	61
ENO1 rescued	660	1,701	2,367	1,231	267	682
ENO1 WT	2,125	3,850	3,349	9,046	1,608	3,587

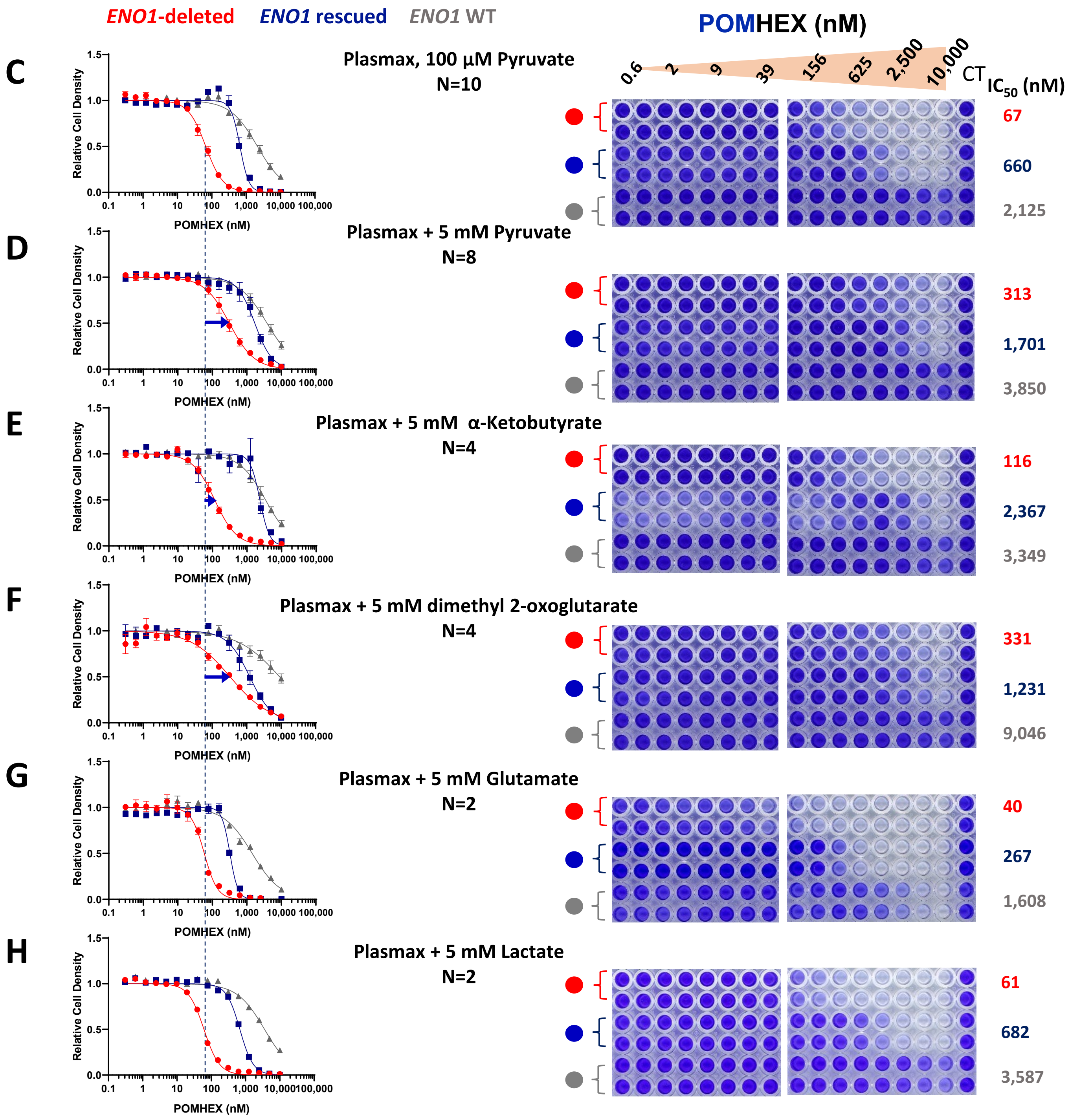

Supplemental Figure S5. **Exogenously supplemented, supraphysiological levels of anaplerotic substrates rescue POMHEX toxicity even in physiological Plasmax^{TM} medium**. **A.** Schematic showing α-ketobutyrate and pyruvate act as electron acceptors from NADH and regenerate NAD+ through the lactate dehydrogenase reaction. Pyruvate, dimethyl 2-oxoglutarate (cell permeable analog of a-ketoglutarate), and glutamate can serve as the sources of carbon atoms for TCA cycle through distinct anaplerotic reactions. Dimethyl 2-oxoglutarate is neutral and cell permeable and enters the TCA cycle rapidly after cleavage by non-specific esterases, while glutamate enters the TCA cycle through the glutamate dehydrogenase or the aminotransferase reactions. **B.** Table representing the IC_{50} values of POMHEX in different Plasmax^{TM} medium supplemented with different anaplerotic substrates. (**C-F**) *ENO1* homozygously deleted (D423; red), *ENO1*-isogenically rescued (D423 *ENO1*; blue), and *ENO1*-WT (LN319; grey) cells were treated with serial dilutions of POMHEX at the indicated doses in Plasmax^{TM} medium (**C**, N=10) or plasmax medium exogenously supplemented with 5 mM pyruvate (**D**, N=8), 5mM α-ketobutyrate (**E**, N=4), 5 mM dimethyl 2-oxoglutarate (**F**, N=4) , 5 mM glutamate (**G**, N=2) and 5mM lactate (**H**, N=2). Following 5-days of drug treatment, cell density was measured by crystal violet staining. Cell density is expressed relative to the DMSO control groups. Where indicated, error bars represent standard error of the mean. IC_{50} of POMHEX for the cell lines in each medium condition is indicated. Blue arrows on the graphs indicate the shift in IC_{50} of POMHEX. Note that pyruvate and dimethyl 2-oxoglutarate provide comparable and maximal rescue to POMHEX treatment**.** These data suggest that pyruvate provides POMHEX toxicity relief primarily by supporting anaplerosis and only minimally by shifting the redox status.

Figure S6

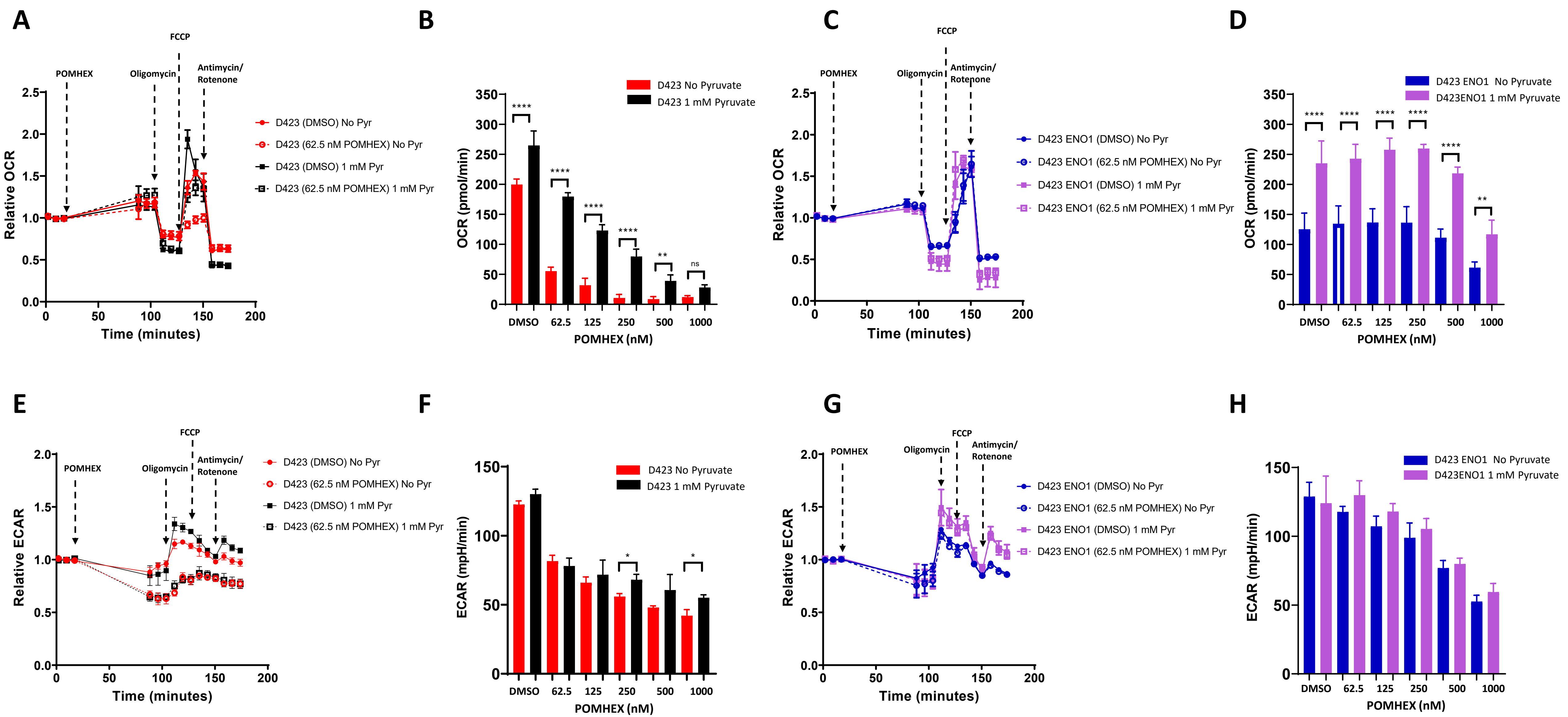
A
POMHEX
Oligomycin
FCCP
Antimycin/ Rotenone
Relative OCR
Time (minutes)
D423 (DMSO) No Pyr
D423 (62.5 nM POMHEX) No Pyr
D423 (DMSO) 1 mM Pyr
D423 (62.5 nM POMHEX) 1 mM Pyr
B
D423 No Pyruvate
D423 1 mM Pyruvate
OCR (pmol/min)
POMHEX (nM)
DMSO
62.5
125
250
500
1000
ns
C
D423 ENO1 (DMSO) No Pyr
D423 ENO1 (62.5 nM POMHEX) No Pyr
D423 ENO1 (DMSO) 1 mM Pyr
D423 ENO1 (62.5 nM POMHEX) 1 mM Pyr
D
D423 ENO1 No Pyruvate
D423ENO1 1 mM Pyruvate
E
Relative ECAR
F
ECAR (mpH/min)
G
H

Supplemental Figure S6. **POMHEX treated cells are substrate limited for mitochondrial respiration which is attenuated by exogenous pyruvate**. **A.** Seahorse Mito Stress Assay on cells treated with POMHEX with or without exogenous supplementation of 1 mM pyruvate. Arrows indicate the sequential injections of POMHEX, oligomycin, carbonyl cyanite-4 (trifluoromethoxy) phenylhydrazone (FCCP), and Rotenone/Antimycin into the wells at the indicated times. **A,C.** Relative oxygen consumption rate of the *ENO1* deleted (**A**) and *ENO1* reconstituted (**B**) cells treated with DMSO or POMHEX (representative 62.5 nM) in Seahorse XF medium with and without 1 mM pyruvate. Data expressed is relative to the baseline OCR. **E,G.** Relative extracellular acidification rate of the cells treated with DMSO or POMHEX (representative 62.5 nM) with and without pyruvate. Data expressed is relative to the baseline ECAR. **B,D.** Maximal respiration in *ENO1* deleted (**B**) or *ENO1* rescued cells(**D**) treated with serial dilutions of POMHEX in Seahorse XF medium without or with 1 mM sodium pyruvate supplemented exogenously. **F,H.** ECAR in *ENO1* deleted (**F**) or *ENO1* reconstituted (**H**) cells treated with serial dilutions of POMHEX in Seahorse medium without or with 1 mM sodium pyruvate supplemented exogenously. Mean and standard error of mean are shown, and **** indicate statistical significance ($p<0.001$) achieved by student's t-test.

Figure S7

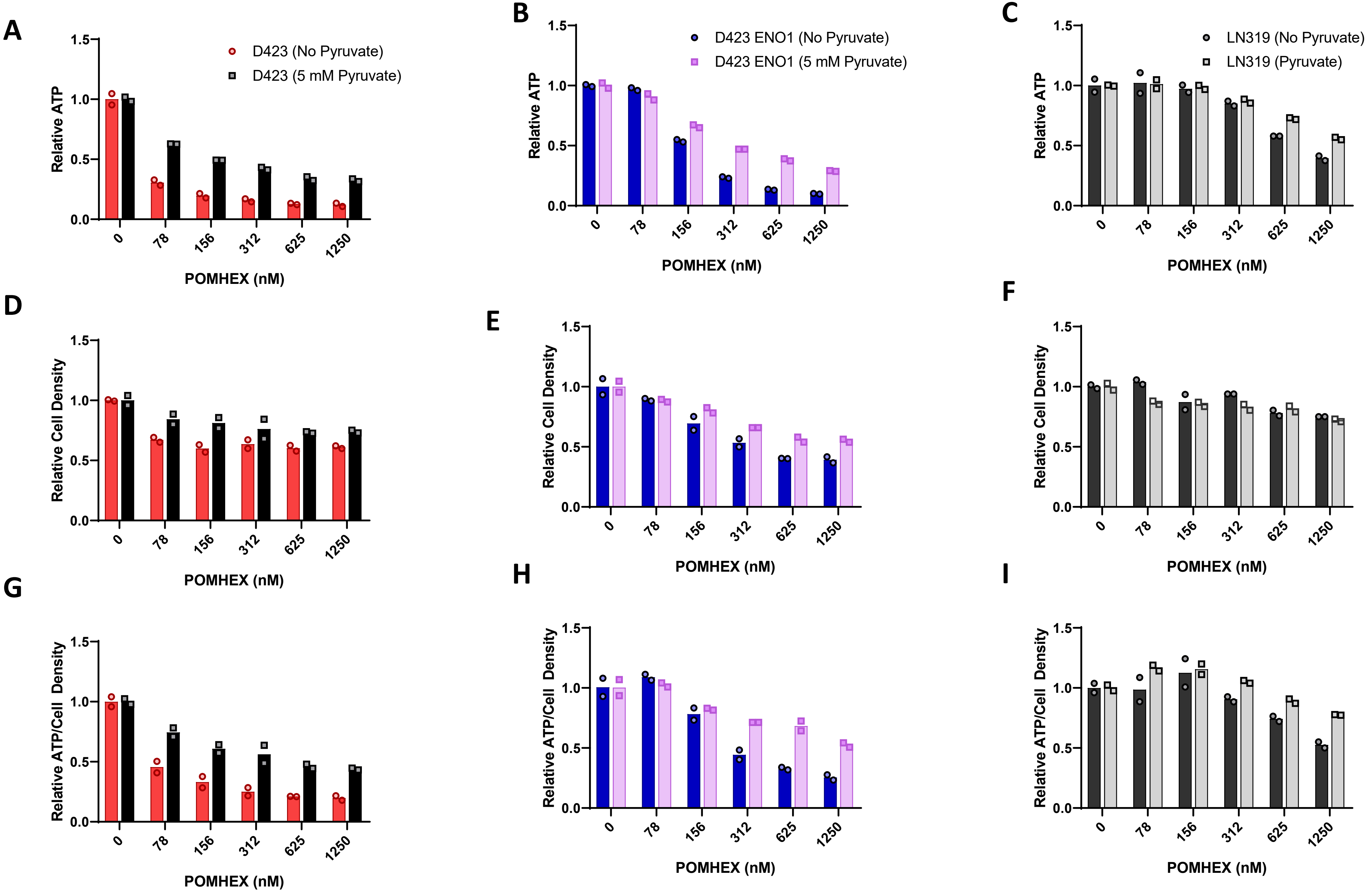

A
D423 (No Pyruvate)
D423 (5 mM Pyruvate)
Relative ATP
POMHEX (nM)
B
D423 ENO1 (No Pyruvate)
D423 ENO1 (5 mM Pyruvate)
Relative ATP
POMHEX (nM)
C
LN319 (No Pyruvate)
LN319 (Pyruvate)
Relative ATP
POMHEX (nM)
D
Relative Cell Density
POMHEX (nM)
E
Relative Cell Density
POMHEX (nM)
F
Relative Cell Density
POMHEX (nM)
G
Relative ATP/Cell Density
POMHEX (nM)
H
Relative ATP/Cell Density
POMHEX (nM)
I
Relative ATP/Cell Density
POMHEX (nM)
0.0
0.5
1.0
1.5
0
78
156
312
625
1250

Supplemental Figure S7. **Enolase inhibition induces bioenergetic stress preceding cell killing, which is rescued by exogenous pyruvate.** *ENO1* homozygously deleted (**A.** D423, N=2), *ENO1*-isogenically rescued (**B.** D423 *ENO1,* N=2), and *ENO1*-WT (**C.** LN319; N=2) cells were treated in serial dilutions of POMHEX at the indicated doses in regular Plasmax medium or Plasmax medium supplemented with 5 mM pyruvate. After 24 hours, CellTitre-Glo® assay was performed to quantify total ATP levels in cells and crystal violet assay was performed to quantify total cell density. Relative ATP levels (**A-C**), relative cell densities (**D-F**) and relative ratio of ATP and cell densities (**G-I**) are expressed. Note that the depletion in ATP levels precedes cell killing by POMHEX, and the effect of POMHEX on ATP levels is partially rescued by addition of supraphysiological levels of pyruvate (5mM). Note that absolute ATP levels were universally higher in pyruvate supplemented media; this is not evident in this graph since the results were normalized to vehicle treated cells.

Figure S8

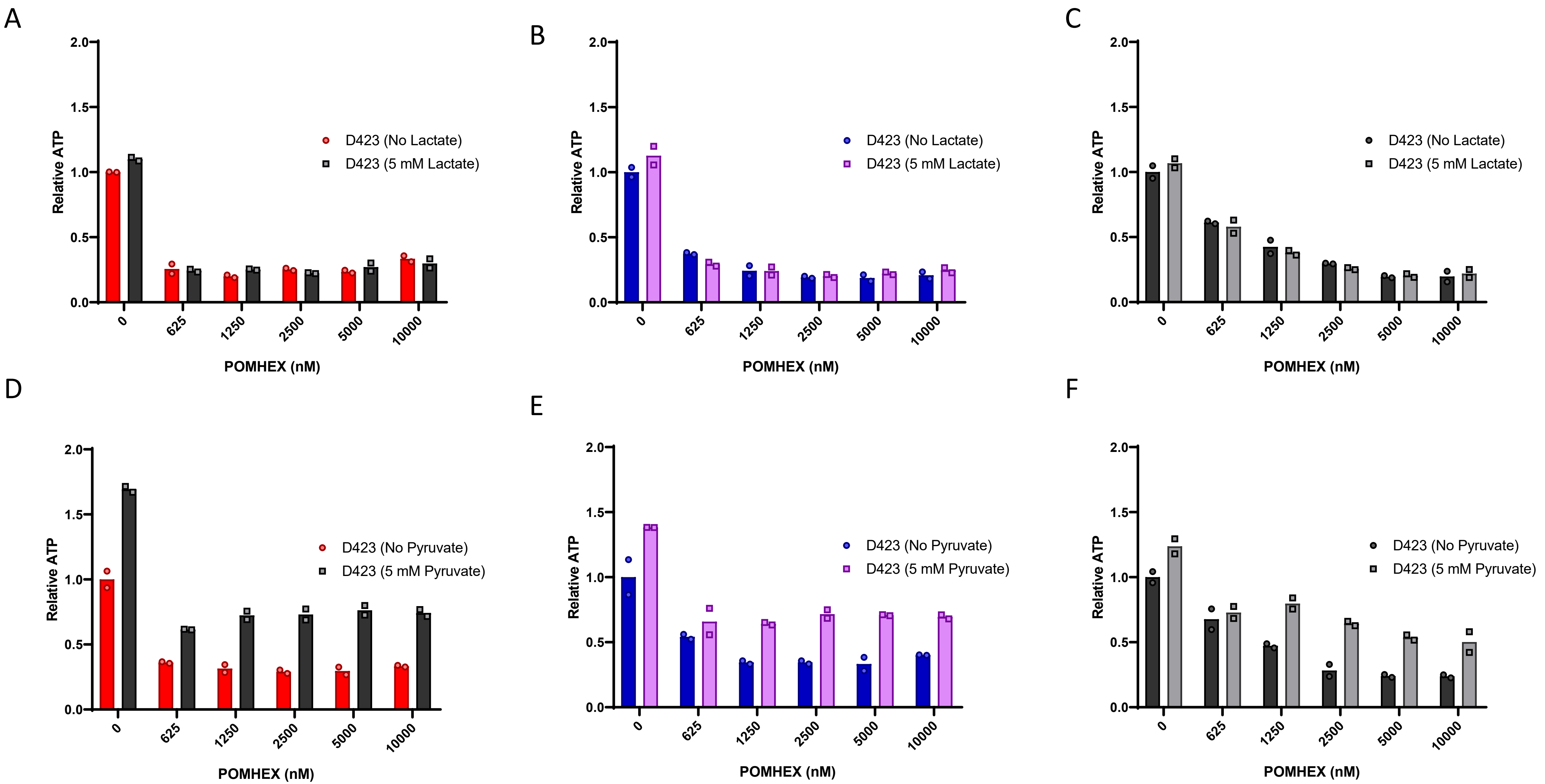
A
B
C
D
E
F
Relative ATP
POMHEX (nM)
0.0
0.5
1.0
1.5
2.0
0
625
1250
2500
5000
10000
D423 (No Lactate)
D423 (5 mM Lactate)
D423 (No Pyruvate)
D423 (5 mM Pyruvate)

Supplemental Figure S8. **Exogenous pyruvate but not lactate rescues ATP production inhibited by enolase inhibitor treatment**. *ENO1* homozygously deleted (**A.** D423, N=2), *ENO1*-isogenically rescued (**B.** D423 *ENO1,* N=2), and *ENO1*-WT (**C.** LN319; N=2) cells were treated in serial dilutions of POMHEX at the indicated doses in regular Plasmax medium or Plasmax medium supplemented with 5 mM lactate or pyruvate. After 24 hours, CellTitre-Glo® assay was performed to quantify total ATP levels in cells and crystal violet assay was performed to quantify total cell density. Relative ATP levels in with or without lactate supplementation (**A-C**) and with or without pyruvate (**D-E**) are shown. Note that the depletion in ATP levels caused by POMHEX treatment is rescued only by pyruvate and not by lactate, indicating that lactate is not an optimal mitochondrial respiration substrate.

Figure S9

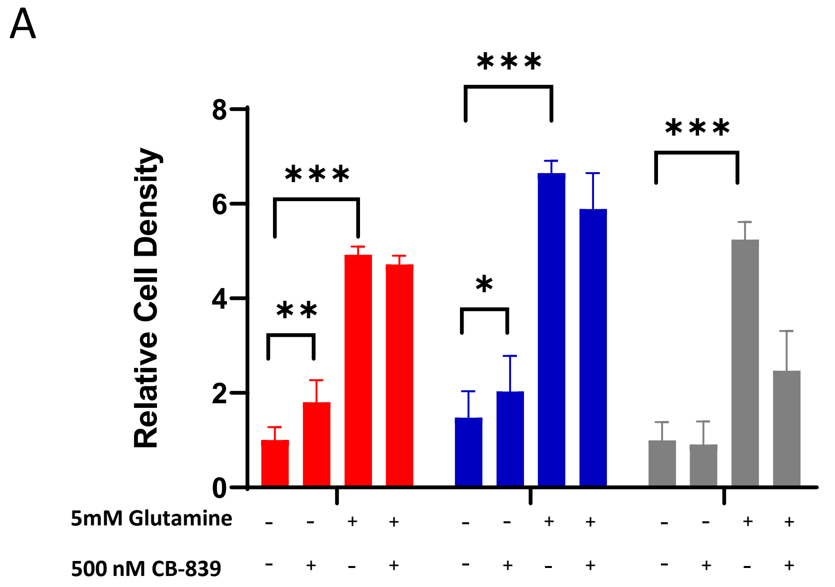
A
***
***
***
**
*
8
6
4
2
0
Relative Cell Density
5mM Glutamine
- - + + - - + + - - + +
500 nM CB-839
- + - + - + - + - + - +

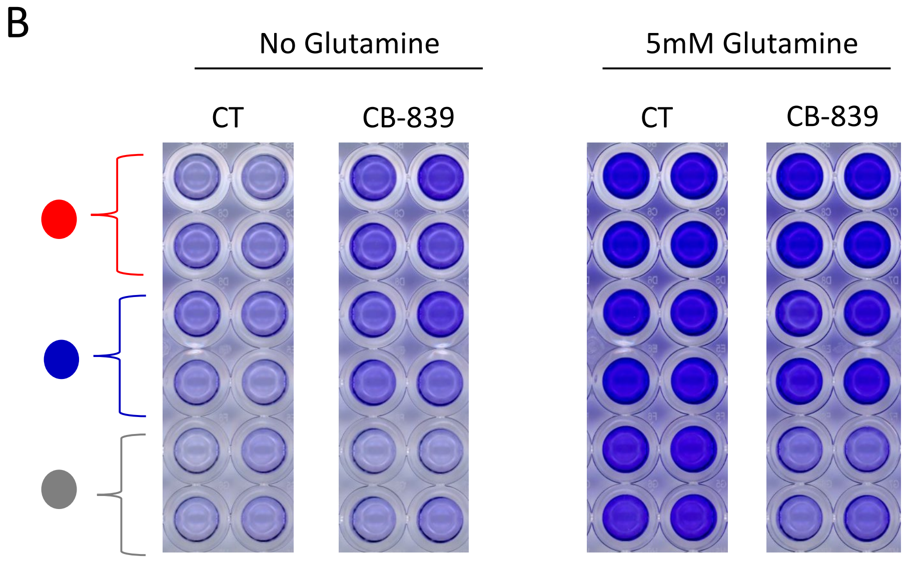
B
No Glutamine
5mM Glutamine
CT
CB-839
CT
CB-839

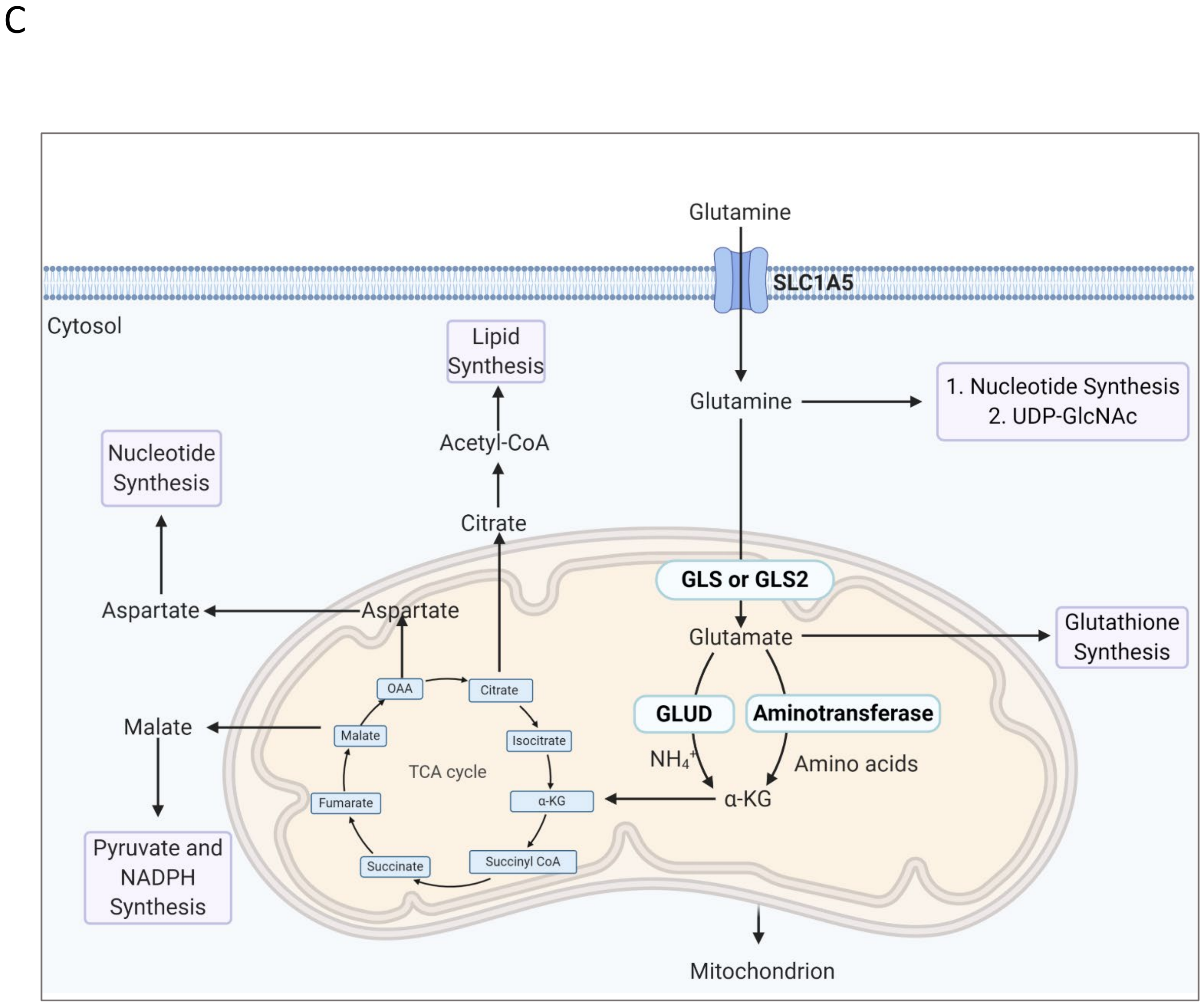
C
Glutamine
SLC1A5
Cytosol
Lipid Synthesis
Glutamine
1. Nucleotide Synthesis
2. UDP-GlcNAc
Acetyl-CoA
Nucleotide Synthesis
Citrate
GLS or GLS2
Aspartate
Aspartate
Glutamate
Glutathione Synthesis
OAA
Citrate
Malate
Malate
Isocitrate
GLUD
Aminotransferase
TCA cycle
NH4+
Amino acids
Fumarate
α-KG
α-KG
Succinate
Succinyl CoA
Pyruvate and NADPH Synthesis
Mitochondrion

Supplemental Figure S9. **Glioma cells exhibit glutamine auxotrophy *in vitro*.** **A.** *ENO1* homozygously deleted (D423,red, N=4), *ENO1*-isogenic rescue (D423 *ENO1*, blue N=4), *ENO1* wild type (LN319, grey, N=4) were grown in glutamine free DMEM and DMEM exogenously supplemented with 5mM glutamine and cell growth as a function of glutamine availability in the medium was measured by crystal violet staining on day 6. Cells were also treated with the glutaminase inhibitor CB-839 (500 nM) in each medium condition. Error bars indicate the standard deviation of the mean, while (***) indicate statistical significance ($p<0.05$) determined by two-way ANOVA and Tukey's HSD post-hoc analysis. **B.** Schematic showing the non-anaplerotic and anaplerotic roles of glutamine. Glutamine is imported into the cells through SLC1A5 or ASCT2. Glutamine is then broken down for biosynthesis of nucleotides or uridine diphosphate-*N*-acetylglucosamine in the cytosol , or is converted to glutamate in the mitochondria by the enzyme glutaminase (GLS or GLS2). Glutamate serves as a precursor for glutathione biosynthesis and can also be converted to α -ketoglutarate (α -KG) by the activity of glutamate dehydrogenase (GLUD1 or GLUD2) or aminotransferases. GLUD reactions yield $NH4+$, while aminotransferase reactions yield amino acids as by-products. α-KG derived from glutamine serves as an anaplerotic substrate for the TCA cycle that generates biosynthetic intermediates as well as reducing equivalents.

Figure S10

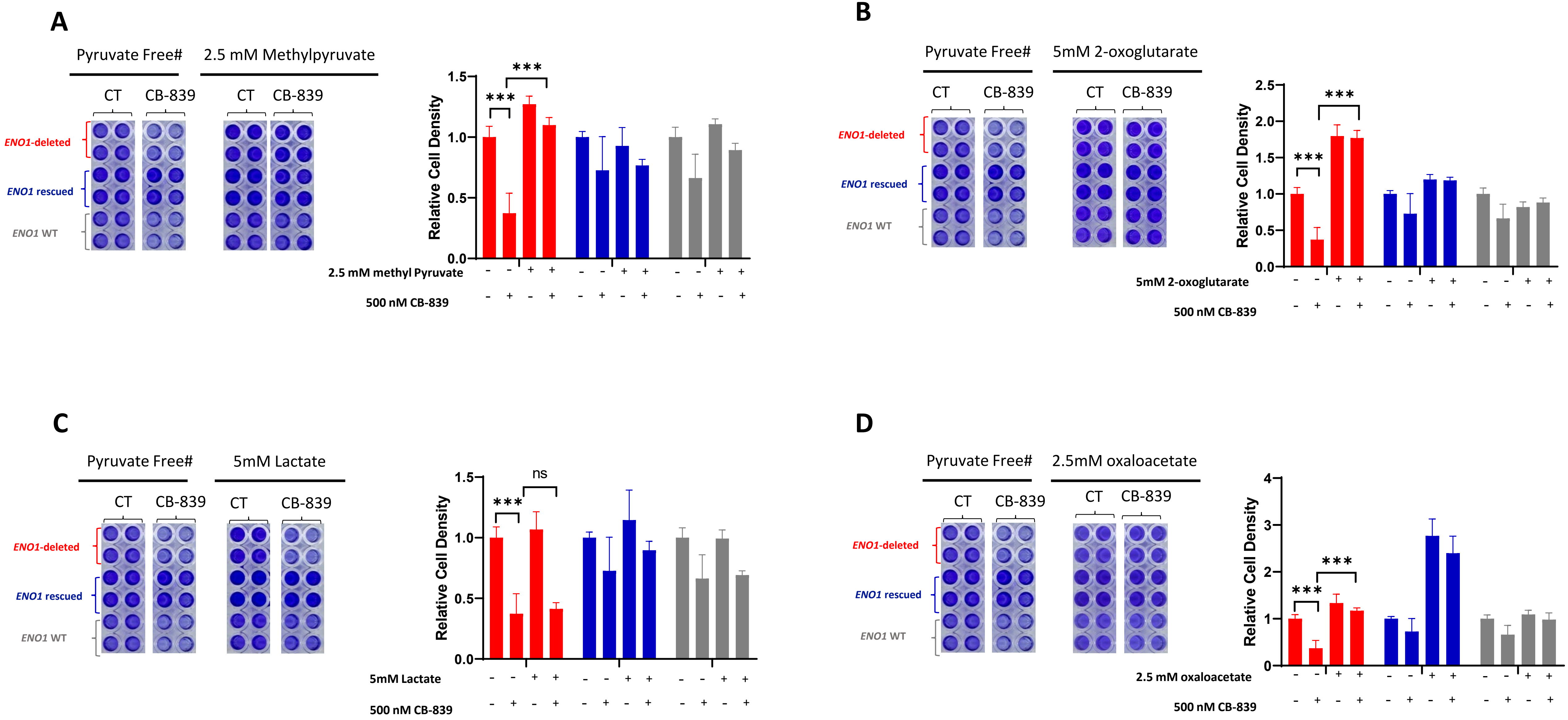
A
Pyruvate Free#
2.5 mM Methylpyruvate
CT
CB-839
ENO1-deleted
ENO1 rescued
ENO1 WT
Relative Cell Density
***
2.5 mM methyl Pyruvate
500 nM CB-839
B
Pyruvate Free#
5mM 2-oxoglutarate
5mM 2-oxoglutarate
C
Pyruvate Free#
5mM Lactate
ns
5mM Lactate
D
Pyruvate Free#
2.5mM oxaloacetate
2.5 mM oxaloacetate

Supplemental Figure S10. **CB-839 toxicity is exaggerated under pyruvate free conditions and reversed by the addition of anaplerotic substrates.** *ENO1* homozygously deleted (D423,red), *ENO1*-isogenic rescue (D423 *ENO1*, blue), and *ENO1* wild type (LN319, grey) cells were grown in pyruvate free (N=32) DMEM or DMEM supplemented with 2.5 mM methyl pyruvate (**A**, N=8), 5mM dimethyl 2-oxoglutarate (**B**, N=8), 5mM Lactate (**C**, N=8) and 2.5 mM oxaloacetate (**D**, N=8). DMSO vehicle or 500 nM CB-839 was administered to the cells and growth inhibition in each medium condition was determined by crystal violet staining after 5 days of drug exposure. Cell density is expressed relative to vehicle control in pyruvate free medium. Mean and +/- S.D. are shown and (***) indicate statistical significance ($p<0.0001$) determined by two-way ANOVA and Tukey's HSD post-hoc analysis. # indicates that the same pyruvate free controls were used for normalization for each anaplerotic substrate.

Figure S11

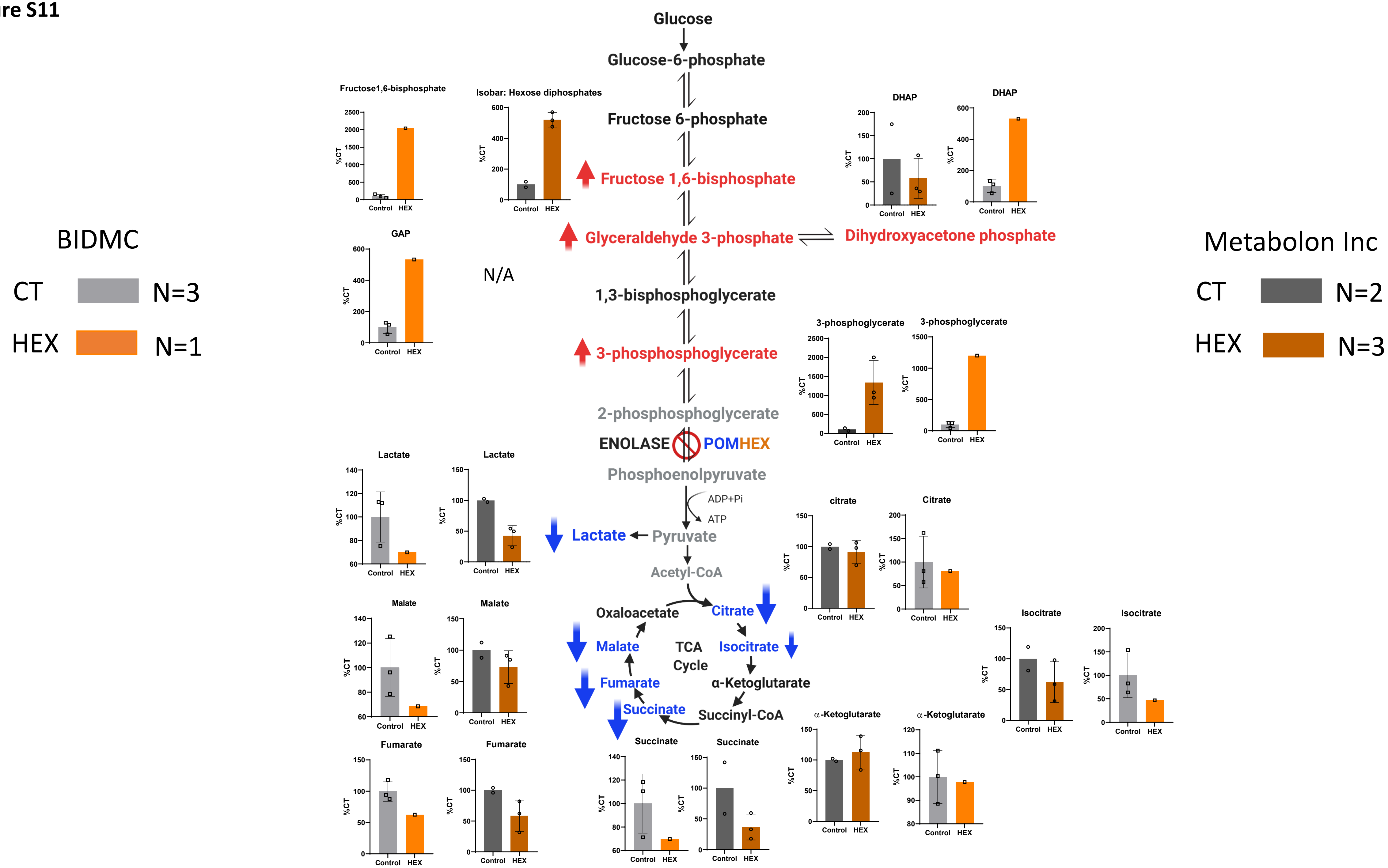
BIDMC
CT
N=3
HEX
N=1
Metabolon Inc
CT
N=2
HEX
N=3
Glucose
Glucose-6-phosphate
Fructose 6-phosphate
Fructose 1,6-bisphosphate
Glyceraldehyde 3-phosphate
Dihydroxyacetone phosphate
1,3-bisphosphoglycerate
3-phosphophoglycerate
2-phosphophoglycerate
ENOLASE
POMHEX
Phosphoenolpyruvate
ADP+Pi
ATP
Lactate
Pyruvate
Acetyl-CoA
Oxaloacetate
Citrate
Malate
TCA
Cycle
Isocitrate
Fumarate
α-Ketoglutarate
Succinate
Succinyl-CoA
N/A
Fructose1,6-bisphosphate
Isobar: Hexose diphosphates
GAP
DHAP
3-phosphoglycerate
Lactate
Malate
Fumarate
Succinate
citrate
Citrate
Isocitrate
α-Ketoglutarate
%CT
Control
HEX

Supplemental Figure S11. **Metabolomic analysis of HEX treated *ENO1* deleted tumors from two different metabolomic platforms confirm elevation of glycolytic metabolites upstream, and reduction in the TCA cycle metabolites, downstream of the enolase reaction**. *ENO1*-deleted (D423) subcutaneous tumors treated with HEX from two independent experiments were subjected to metabolomic analyses using either the BIDMC metabolomic core or the Metabolon Inc metabolomics platform. **A**. For the BIDMC platform, vehicle control group, N=3 and HEX, N=1) and Metabolon Inc platform, (vehicle control group N=2,HEX treated N=3). Mean and +/- S.D. where relevant with individual data points are shown.

Figure S12

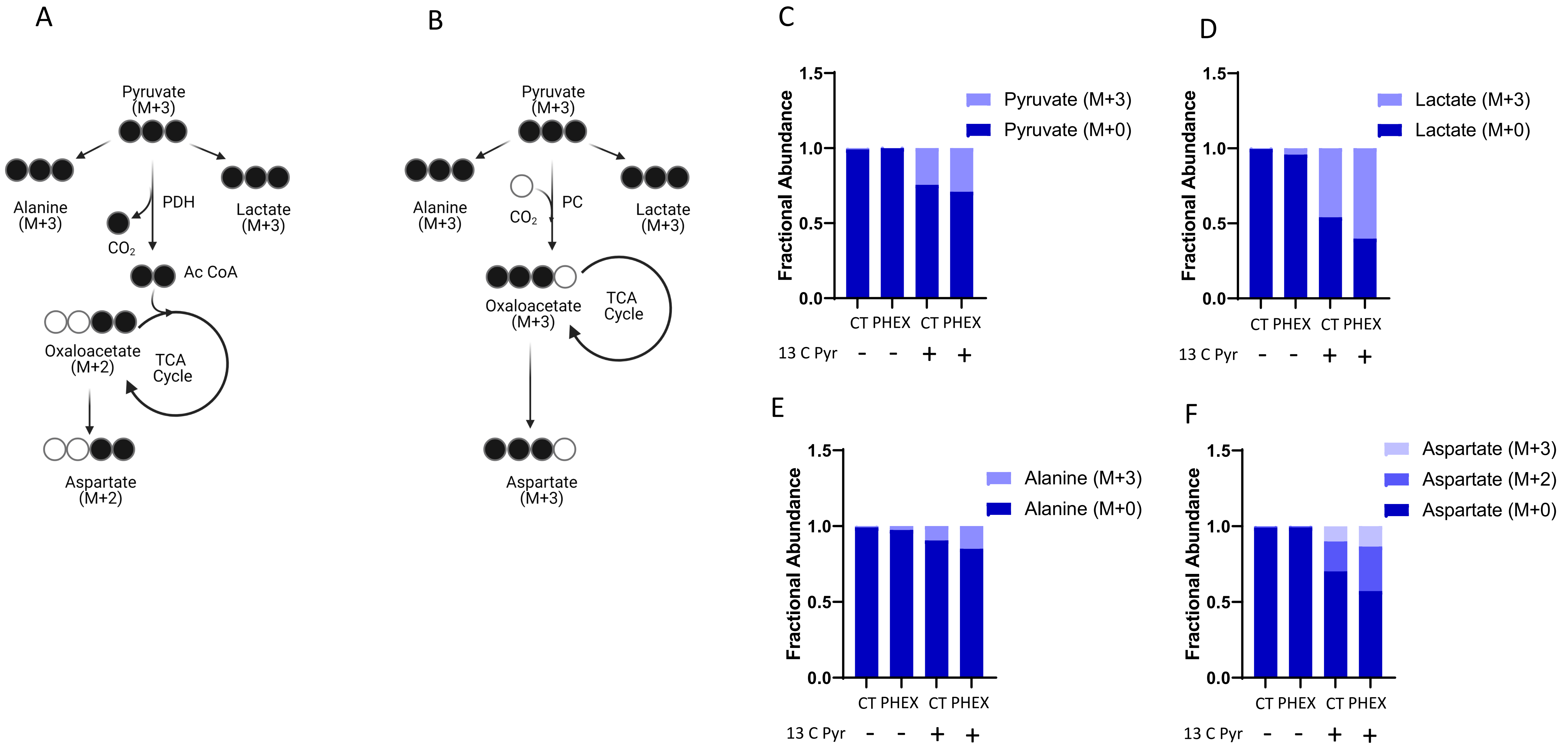
A
Pyruvate (M+3)
Alanine (M+3)
Lactate (M+3)
PDH
CO_2
Ac CoA
Oxaloacetate (M+2)
TCA Cycle
Aspartate (M+2)
B
Pyruvate (M+3)
Alanine (M+3)
Lactate (M+3)
PC
CO_2
Oxaloacetate (M+3)
TCA Cycle
Aspartate (M+3)
C
Fractional Abundance
1.5
1.0
0.5
0.0
Pyruvate (M+3)
Pyruvate (M+0)
CT PHEX CT PHEX
13 C Pyr - - + +
D
Fractional Abundance
1.5
1.0
0.5
0.0
Lactate (M+3)
Lactate (M+0)
CT PHEX CT PHEX
13 C Pyr - - + +
E
Fractional Abundance
1.5
1.0
0.5
0.0
Alanine (M+3)
Alanine (M+0)
CT PHEX CT PHEX
13 C Pyr - - + +
F
Fractional Abundance
1.5
1.0
0.5
0.0
Aspartate (M+3)
Aspartate (M+2)
Aspartate (M+0)
CT PHEX CT PHEX
13 C Pyr - - + +

Supplemental Figure S12. **Exogenously supplemented pyruvate contributes to TCA cycle through both pyruvate carboxylase and pyruvate dehydrogenase reactions**. **A-B.** Schematic of labelling patterns of U-13C pyruvate into the TCA cycle via the pyruvate carboxylase (PDH) (**A**) or pyruvate dehydrogenase (PC) (**B**) reactions. *ENO1* homozygously deleted (D423), cells were grown in pyruvate free DMEM or pyruvate free DMEM supplemented with 5 mM U-13C pyruvate with or without 75 nM POMHEX for 12 hours. Metabolites were extracted in 80% cold methanol and subjected to mass-spec analysis. Fractional abundance of isotopomers of pyruvate, lactate, alanine and aspartate are shown (**C-F**). Note that exogenous pyruvate enters the TCA cycle through both PC and PDH mechanisms and pyruvate supplementation in the context of POMHEX treatment increases both PC and PDH fluxes (**F**).

Figure S13

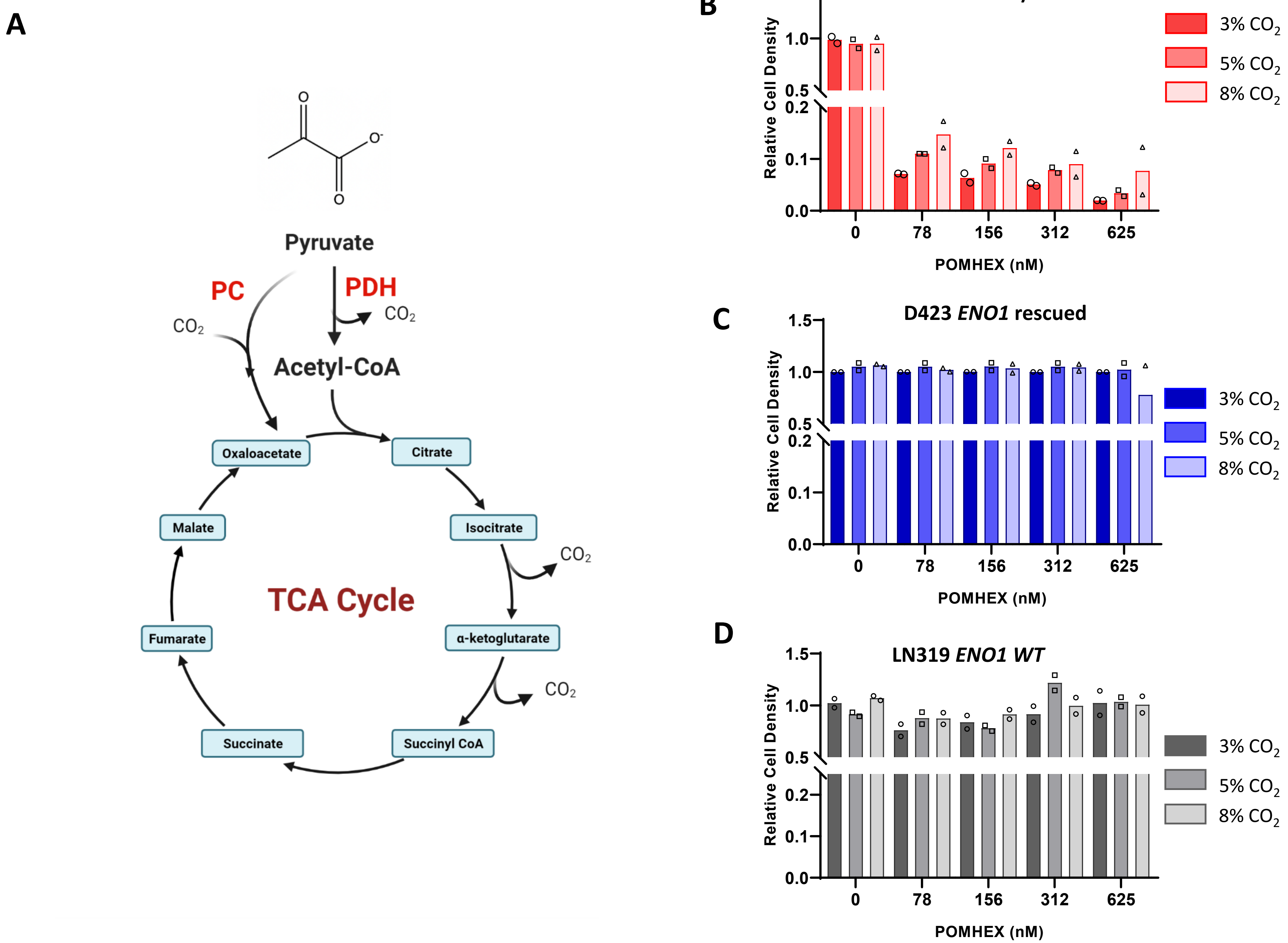
A
O
O-
O
Pyruvate
PC
PDH
CO2
CO2
Acetyl-CoA
Oxaloacetate
Citrate
Isocitrate
CO2
Malate
TCA Cycle
Fumarate
α-ketoglutarate
CO2
Succinate
Succinyl CoA
B
D423 ENO1 -/-
Relative Cell Density
1.5
1.0
0.5
0.2
0.1
0.0
0
78
156
312
625
POMHEX (nM)
3% CO2
5% CO2
8% CO2
C
D423 ENO1 rescued
Relative Cell Density
1.5
1.0
0.5
0.2
0.1
0.0
0
78
156
312
625
POMHEX (nM)
3% CO2
5% CO2
8% CO2
D
LN319 ENO1 WT
Relative Cell Density
1.5
1.0
0.5
0.2
0.1
0.0
0
78
156
312
625
POMHEX (nM)
3% CO2
5% CO2
8% CO2

Supplemental Figure S13. **CO_2 levels modulate POMHEX toxicity. A.** Schematic of pyruvate entry into the TCA cycle via the pyruvate carboxylase (PC) or pyruvate dehydrogenase (PDH) reactions. **C-E.** *ENO1* homozygously deleted (**A.** D423; red, N=2), *ENO1*-isogenically rescued (**B.** D423 *ENO1*; blue, N=2), and *ENO1* WT (**C.** LN319; grey, N=2) cells were treated in regular DMEM with serial dilutions of POMHEX at the indicated doses in 3%, 5% and 8% CO_2. After 5 days, cells were fixed in 10% formalin and the terminal cell density determined by crystal violet staining. The cell densities are expressed relative to untreated control. Consistent with the anaplerotic requirement of CO_2 for pyruvate carboxylase reaction, the toxicity of POMHEX is exacerbated in low CO_2 conditions selectively in *ENO1* deleted cells.

Figure S14

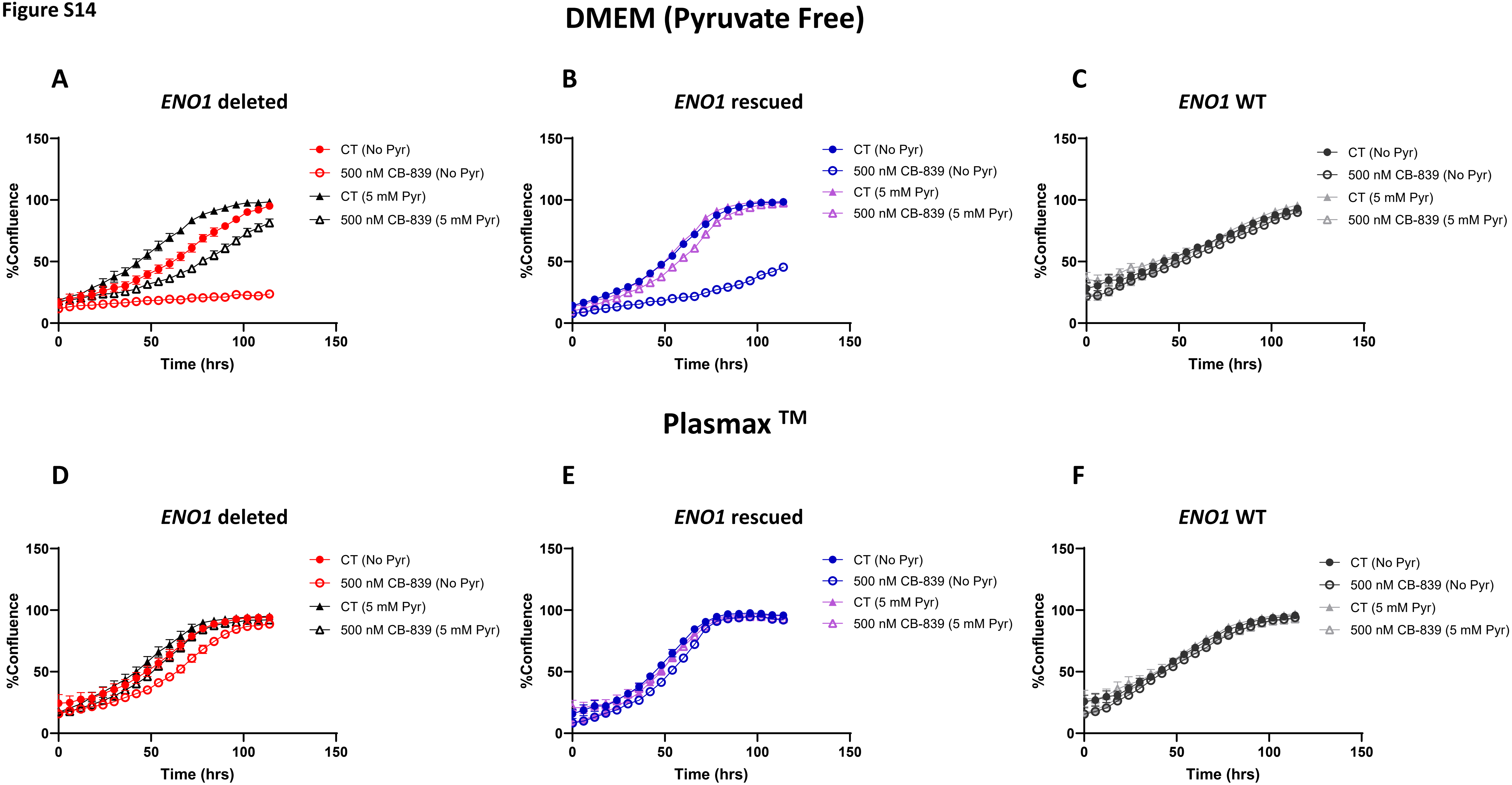

DMEM (Pyruvate Free)
A
ENO1 deleted
B
ENO1 rescued
C
ENO1 WT
Plasmax TM
D
ENO1 deleted
E
ENO1 rescued
F
ENO1 WT
%Confluence
Time (hrs)
0
50
100
150
CT (No Pyr)
500 nM CB-839 (No Pyr)
CT (5 mM Pyr)
500 nM CB-839 (5 mM Pyr)

Supplemental Figure S14. **Sensitivity of glioma cells to CB-839 is attenuated in physiological Plasmax™ medium**. *ENO1* homozygously deleted (D423), *ENO1*-isogenic rescue (D423 *ENO1*), and *ENO1* wild type (LN319) cells were grown in pyruvate free DMEM (**A-C**) or Plasmax™ (**D-F**) medium with or without 5 mM pyruvate supplementation. Cells were treated with 500 nM CB-839 or vehicle DMSO (N=6 in each condition). The growth of the cells over a 4 day period was monitored using Incucyte®. Note that CB-839 toxicity in *ENO1* deleted cells is attenuated in Plasmax™ medium. Exogenous pyruvate supplementation confers additional rescue to CB-839 toxicity in both DMEM and Plasmax™ medium.

Figure S15

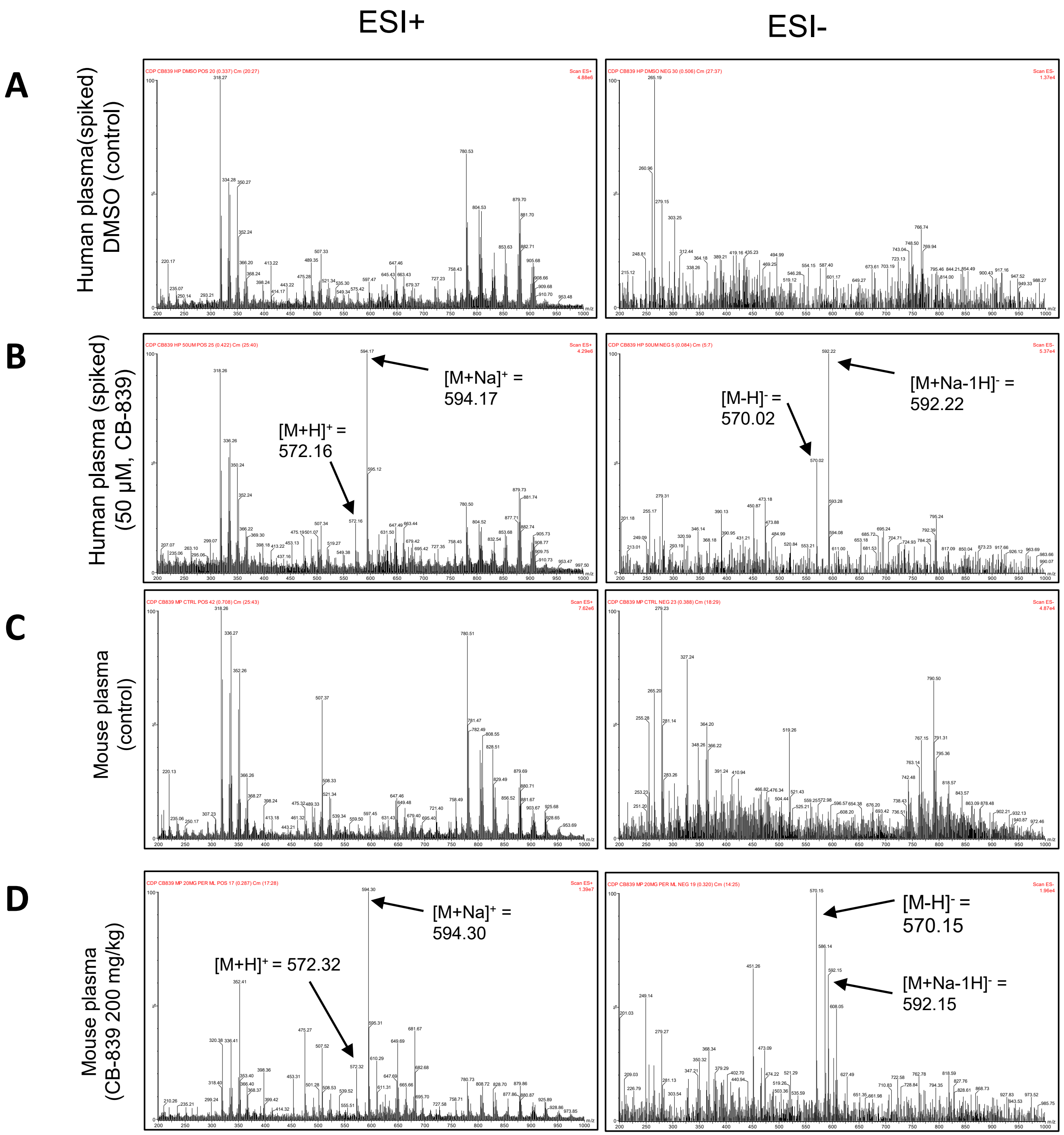
ESI+
ESI-
A
Human plasma(spiked) DMSO (control)
B
Human plasma (spiked) (50 µM, CB-839)
[M+Na]+ = 594.17
[M+H]+ = 572.16
[M-H]- = 570.02
[M+Na-1H]- = 592.22
C
Mouse plasma (control)
D
Mouse plasma (CB-839 200 mg/kg)
[M+Na]+ = 594.30
[M+H]+ = 572.32
[M-H]- = 570.15
[M+Na-1H]- = 592.15

Supplemental Figure S15. **Orally administered CB-839 is detectable in mouse plasma 2 hours post drug administration. via LC-MS (ESI**). (**A-B)** Human plasma was spiked with serial dilutions of CB-839 (positive control) and extracted with one volume of ethyl acetate. Representative 50 µM CB-839 and DMSO control in human plasma are shown.
(**C-D**) Plasma was collected from mice treated with vehicle or CB-839 two hours post drug administration. One volume of ethyl acetate was used to extract CB-839 from mouse plasma and detected using LC-MS (ESI)**.**
